# Measuring Technology-Facilitated Sexual Violence and Abuse: Scoping Review of Existing Measures

**DOI:** 10.2196/90068

**Published:** 2026-04-14

**Authors:** Sharon Hoi Lam Pak, Linruo Zhang, Chanchan Wu, Pui Hing Chau, Caroline Bradbury-Jones, Edmond Pui Hang Choi

**Affiliations:** 1 School of Nursing Li Ka Shing Faculty of Medicine The University of Hong Kong Hong Kong China (Hong Kong); 2 School of Nursing Faculty of Health and Social Science The Hong Kong Polytechnic University Hong Kong China (Hong Kong); 3 Department of Nursing and Midwifery University of Birmingham Birmingham United Kingdom

**Keywords:** image-based sexual abuse, online sexual violence, measures, scoping review, structural topic modeling, technology-facilitated sexual violence

## Abstract

**Background:**

Technology-facilitated sexual violence and abuse (TFSVA) encompasses sexual harms perpetrated through digital means. Rapid developments in this field pose challenges in precisely defining and measuring TFSVA. Consequently, existing measures may fail to capture its multifaceted nature.

**Objective:**

This scoping review aimed to map and appraise current knowledge on measures used to assess TFSVA, specifically to identify and summarize existing measures of TFSVA and its associated constructs, including victimization, perpetration, related attitudes, motivations, and impacts.

**Methods:**

Following the Joanna Briggs Institute framework, we searched 10 English and Chinese databases (PubMed, Embase, CINAHL, Scopus, ProQuest, Web of Science, Chinese National Knowledge Infrastructure, SinoMed, Index to Taiwan Periodical Literature, and Ariti Library) from inception to January 13, 2026. We included peer-reviewed empirical studies focused on developing, adapting, or validating any TFSVA measures across populations and settings. Two independent reviewers screened records and extracted data. Data were analyzed using structural topic modeling (STM) and thematic analysis. Risk-of-bias assessment was not conducted, in accordance with scoping review methodology.

**Results:**

Among the 319 included studies, 218 distinct measures were identified. The analysis revealed a fragmented and conceptually narrow measurement landscape. Most measures focused on specific subdomains, primarily nonconsensual sexting (130/218, 59.6%), with less attention to image-based sexual abuse (30/218, 13.8%), online sexual harassment (27/218, 12.4%), online grooming (13/218, 6.0%), and cyber sexual dating abuse (8/218, 3.7%). Nearly half (108/218, 49.5%) assessed both victimization and perpetration, while 96 out of 218 (44.0%) focused solely on victimization, 12 out of 218 (5.5%) on perpetration, 1 out of 218 (0.5%) on the bystander perspective, and the remaining 1 out of 218 (0.5%) addressed all aspects. The vast majority (199/218, 91.3%) measured behaviors, whereas attitudes, motivations, and impacts were rarely assessed. STM identified 9 key topics, synthesized into 3 core dimensions: consent violation and coercive control; status degradation and demeaning harassment; and contextual and relational framing. Thematic analysis highlighted 5 themes: TFSVA acts, attitudes and beliefs, motivational factors, tactical and contextual dynamics, and survivor impact. Integrating these findings reveals a fundamental conceptual misalignment between existing measures and the full scope of TFSVA.

**Conclusions:**

This first comprehensive synthesis of existing TFSVA measures innovatively integrates STM and thematic analysis. While the absence of a risk-of-bias assessment precludes definitive conclusions regarding measurement robustness, this review moves beyond a descriptive catalog to identify substantive gaps within TFSVA-specific measures, most notably the omission of artificial intelligence–facilitated tactics, intersectional vulnerabilities, and institutional factors. These findings provide an empirical basis for developing robust, culturally sensitive measures essential for targeted research, effective policy, and responsive social support services in a rapidly evolving digital landscape.

**Trial Registration:**

Open Science Framework 10.17605/OSF.IO/Q5ETW; https://doi.org/10.17605/OSF.IO/Q5ETW

## Introduction

### Rationale

Technology-facilitated sexual violence and abuse (TFSVA) is a pervasive and urgent societal challenge, representing a convergence of digital innovation and sexual violence. Defined as any sexual violence and abuse perpetrated or amplified through digital technologies—from smartphones and social media platforms to artificial intelligence (AI)–driven applications and GPS tracking—TFSVA encompasses a spectrum of harms, including image-based sexual abuse (IBSA), cyberstalking, and targeted online sexual harassment [1,2]. Specific manifestations range from the nonconsensual sharing of intimate content to the doxing of sensitive personal information, such as sexual orientation or sexually transmitted infection status [3]. This review uses TFSVA as the overarching term for these technology-mediated sexual harms [3].

The rapid proliferation of digital technologies has precipitated a rise in TFSVA worldwide, with studies indicating that 16.6%-20% of adults in the United Kingdom and Australia have reported online sexual harassment in their lifetime, disproportionately affecting women and youth [4-6]. A meta-analysis and systematic review of 25 studies found that 8.8% of individuals have experienced nonconsensual sharing of sexual images or sexually explicit messages (sexting), with many facing threats or unauthorized image capture [7]. TFSVA produces severe consequences, including anxiety, depression, posttraumatic stress disorder, and increased suicidal risk, often stemming from profound privacy violations, stigma, and blaming the individual [1,8]. The pervasive and enduring nature of these digital harms underscores the urgent need for effective identification methods and comprehensive intervention strategies.

A robust and comprehensive measure of TFSVA is critical for understanding its prevalence, dynamics, and impact, as well as for informing prevention, intervention, and policy. However, the landscape of TFSVA is complex and rapidly evolving [6]. This poses a significant challenge in defining and categorizing TFSVA, and existing measures often struggle to capture its multifaceted and context-specific nature [6,7]. One of the primary difficulties in addressing this issue lies in the absence of a universally agreed-upon terminology or definition to describe the myriad ways in which technology is used to perpetrate acts of sexual abuse [6,7]. The absence of shared definitions can lead to underrecognition and underreporting of TFSVA [9], impeding accurate estimation of prevalence and effects [10]. Additionally, the lack of standardized definitions and measures can complicate cross-study comparisons and the aggregation of data [11]. Therefore, it is important to continue gathering data on whether this field of research is reaching a consensus on developing a uniform and comprehensive measure.

To the best of our knowledge, no prior review has systematically mapped existing TFSVA measures. One explanation for this absence is that conducting a systematic review on this topic is challenging due to the lack of precise definitions and standardized research variables in the current evidence literature. This has led to a wide array of measures being used to assess different forms and outcomes of TFSVA. Given these challenges, a scoping review is the most suitable approach for examining emerging evidence before conducting a systematic review [12]. A scoping review is particularly useful for mapping existing knowledge on a topic based on the available literature, thereby providing an overview of the current state of research and identifying gaps in the evidence [13]. It is a transparent and increasingly recognized methodology that can address a broad range of research questions and identify evidence gaps [13]. Although the concept of TFSVA is not yet well defined, to allow for a comprehensive exploration of the topic, TFSVA in this review refers to any form of sexual violence, exploitation, or harassment through the misuse of digital technologies [3].

### Objectives

This scoping review aimed to map and appraise current knowledge on measures used to assess TFSVA. The primary question guiding the review was “What measures are currently available to assess TFSVA?” Subquestions addressed measurement domains, psychometric properties, target populations, and contextual applications to ensure comprehensive coverage of the field. The objectives were to (1) identify and summarize existing measures for TFSVA and its associated constructs, including experiencing harm, perpetration, related attitudes, motivations, and impacts; (2) clarify key concepts and definitions of TFSVA in the literature; (3) evaluate the applicability and validity of these measures across different populations and settings; and (4) identify content coverage and gaps in current measurement approaches.

## Methods

### Protocol and Registration

The scoping review was conducted according to the 5 standard stages of scoping review methodology outlined in the a priori protocol [14], which was registered with the Open Science Framework [15] on March 13, 2024. The review adhered to Joanna Briggs Institute guidance and the PRISMA-ScR (Preferred Reporting Items for Systematic Reviews and Meta-Analyses Extension for Scoping Reviews) reporting standards [12,16]. Deviations from the published protocol were explicitly noted in the relevant sections below.

### Eligibility Criteria

The screening of studies was guided by the Population-Concept-Context (PCC) framework [17]. The core inclusion criteria were as follows: (1) any population, including survivors, perpetrators, and bystanders, without restriction on sexual orientation, gender, age, or ethnicity; (2) empirical studies focused on the development, cultural adaptation, or validation of a measure for TFSVA; and (3) any geographical, cultural, or online setting. All peer-reviewed journal articles in English or Chinese that reported quantitative, qualitative, or mixed methods empirical data were included. Studies that described technology misuse without a sexual or intimate focus were excluded. For this review, a “measure” was defined as any instrument, tool, or technique used to assess, quantify, or characterize TFSVA or its associated constructs. This included multiitem scales, single-item questions, qualitative questions, and adapted or modified versions of existing measures. No minimum number of items was required for inclusion. Both validated and author-developed measures used in empirical studies were eligible.

### Information Sources

The literature search and its reporting followed the PRISMA-S (extension to the PRISMA Statement for Reporting Literature Searches in Systematic Reviews) guidelines [18]. A systematic search was conducted across 10 databases from database inception to January 13, 2026, including 6 English-language electronic databases (PubMed, Embase, CINAHL, Scopus, ProQuest, and Web of Science) and 4 Chinese databases (Chinese National Knowledge Infrastructure, SinoMed, Index to Taiwan Periodical Literature, and Airiti Library). Study registries were not searched, as the inclusion criteria focused exclusively on published peer-reviewed studies. Registries primarily contain protocols, preprints, and trial records, which were not eligible for inclusion. Online resources and browsing were not conducted, as database searching comprehensively covered the published literature and was supplemented by citation searching of included studies. No other search methods were used beyond the database and citation searches described.

### Search

The search strategy combined keywords and controlled vocabulary terms, such as Medical Subject Headings, for the key concepts of the PCC framework. Search filters were not applied to maintain search sensitivity. The search strategy relied on controlled vocabulary and keywords only.

Searches were limited to English- and Chinese-language peer-reviewed empirical studies. To ensure the review reflects the most current literature, the search was executed in 2 phases. The original search, detailed in the published protocol [14], covered records from database inception to December 31, 2024. An updated search was conducted using an identical strategy to capture records published from January 1, 2025, to January 13, 2026. As this was not an update of a prior review, search updates were not applicable. The full electronic search strategy for all databases is provided in Multimedia Appendix 1. To identify additional relevant papers, the reference lists of included studies were hand-searched. We did not contact individual researchers, experts, or organizations for unpublished measures or gray literature, as this scoping review aimed to map the publicly available published literature rather than solicit unpublished measures.

### Selection of Sources of Evidence

Following the search, all records were collated and managed using EndNote 20 (Clarivate Plc). No other systematic review software was used for screening or data extraction. After automatic duplicate removal using EndNote, a manual check was performed. Two reviewers independently screened titles, abstracts, and full texts against the inclusion criteria. Any disagreements were resolved through consensus or consultation with a third researcher. Studies excluded at the full-text review stage were documented with specific reasons, aligned with the PCC framework.

### Data Items and Data Charting Process

Data were extracted using a comprehensive data charting form detailed in the published protocol [14]. The form captured variables across several domains aligned with the PCC framework: (1) study identification and design; (2) population characteristics (eg, age group, sexual orientation, role as survivor, perpetrator, or bystander); (3) geographical setting; (4) the specific type and definition of TFSVA measured; (5) details of the measure used, including its name, language, and reported psychometric properties; and (6) key outcomes related to TFSVA.

The extraction process began with a pilot phase, during which 2 reviewers independently extracted data from 5 studies to establish consensus and refine the form. The remaining data were extracted by 1 reviewer and confirmed by a second reviewer. Discrepancies were resolved through discussion, and a third reviewer was consulted when necessary.

### Synthesis of Results

#### Overview

Our analysis followed a mixed methods design to integrate quantitative and qualitative insights. This was implemented through a 2-stage process: (1) a descriptive synthesis guided by the PAGER (Patterns, Advances, Gaps, Evidence for practice, and Research Recommendations) Framework to generate higher-level insights [19]; and (2) a content analysis using both structural topic modeling (STM) [20] and reflexive thematic analysis.

#### Descriptive Synthesis Using the PAGER Framework

The PAGER Framework provided a structured approach to synthesize and report findings [19]. We applied this framework to systematically categorize the descriptive characteristics of the included studies and their measures across 5 dimensions:

- Patterns: identifying dominant trends in the literature.
- Advances: highlighting methodological and conceptual progress.
- Gaps: recording underrepresented populations, contexts, or TFSVA domains.
- Evidence for practice: distilling findings applicable to current research and policy.
- Research recommendations: formulating priorities for future measure development.

#### Analysis of Measure Content

To analyze the substantive content of the measures, STM and reflexive thematic analysis were conducted on the same set of questionnaire items. This approach aligns with the methodological principle that qualitative and computational text analyses are complementary and analogous processes for identifying patterns in textual data [21]. STM provided a quantitative and reproducible overview of latent conceptual patterns across the full dataset, while reflexive thematic analysis offered qualitative depth, capturing detailed meaning, contextual nuances, and specific question types that purely frequency-based analysis might overlook.

STM is a text-mining technique that identifies latent topics within a text corpus. Each questionnaire item was treated as a separate document. Text preprocessing included lowercasing, removal of punctuation and digits, whitespace normalization, lemmatization, synonym mapping (eg, “photos or images or pics” → picture), and stop-word removal. We fitted STM models with the number of topics (K) ranging from 3 to 10, evaluating model fit using held-out likelihood, residuals, lower bound, semantic coherence, and exclusivity. After identifying K=7, 9, and 10 as offering the best balance of fit, we estimated unsupervised STMs (without covariates) and selected K=9 for final reporting based on balanced fit and conceptual interpretability. All analyses were conducted in R (R Foundation) using the stm (modeling and diagnostics), ggplot2 (visualization), and textstem (lemmatization) packages. This analysis yielded a quantitative map of the dominant conceptual patterns across all measurement items. Interpretive synthesis was performed to translate statistical topics into higher-order conceptual dimensions.

Reflexive thematic analysis was conducted following the 6-phase approach by Clarke and Braun [22]: (1) familiarization with the measurement items; (2) initial code generation; (3) theme search; (4) theme review; (5) theme definition and naming; and (6) report production. Analytical memos documented interpretive decisions and theoretical insights throughout, ensuring thematic robustness and grounding in the data. This independent analysis provided rich, detailed descriptions of question types, contextual nuances, and underlying constructs, complementing and contextualizing the broader statistical patterns identified by STM.

The findings from these 2 analyses were integrated to identify gaps and misalignment within the TFSVA measurement field. Consistent with scoping review methodology, risk-of-bias assessment was omitted [16], although only peer-reviewed articles were included to maintain quality standards.

### Ethics Considerations

This scoping review involved a secondary analysis of previously published, publicly available literature. All included studies reported obtaining ethical approval from their respective institutional review boards or equivalent bodies, as documented in the original publications. No additional ethical approval was required for this review, as it did not involve primary data collection from human participants.

## Results

### Overview of Included Studies and TFSVA Measures

**Figure 1:**
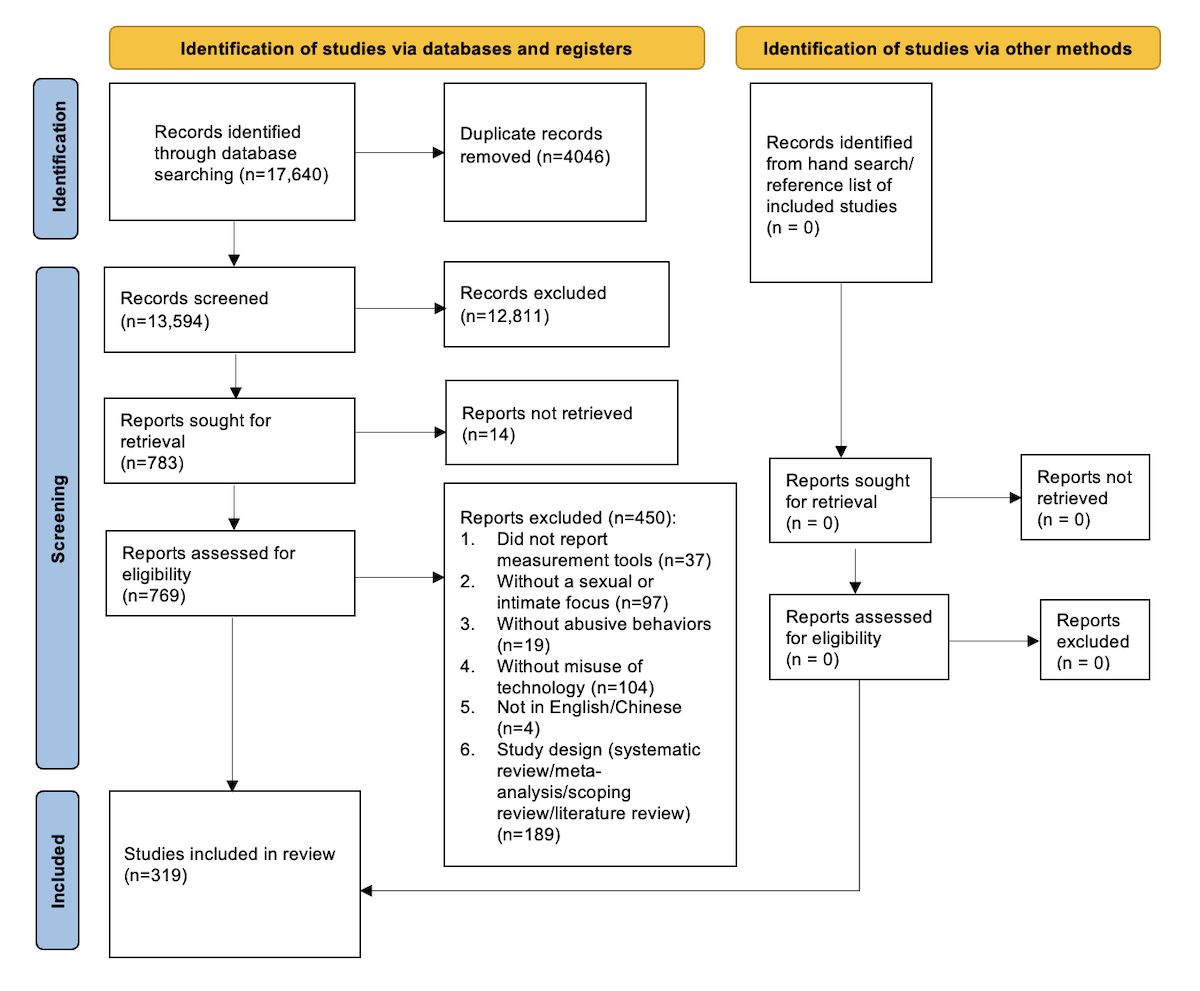
PRISMA (Preferred Reporting Items for Systematic Reviews and Meta-Analyses) flow diagram of study selection. This diagram shows the number of records identified, screened, assessed for eligibility, and included in the scoping review of technology-facilitated sexual violence and abuse measures, from database searching through final inclusion.

The database searches yielded a total of 17,640 studies. After removing 4046 duplicates, 13,594 references remained for further screening. Specifically, 12,811 records were excluded after title and abstract screening. Of the 783 remaining records for full-text screening, 14 were not retrieved and 450 were excluded for specific reasons. Finally, 319 studies were included in the analysis. From the perspective of publication language, all included studies were in English, and no relevant studies were published in Chinese. Study screening and selection were conducted following the PRISMA flow diagram [23]. The detailed study selection process and specific reasons for exclusion are presented in Figure 1. Also see Multimedia Appendices 2 and 3 for PRISMA-ScR and PRISMA-S checklists, respectively.

Included studies were published from September 2011 to January 2026 (Multimedia Appendix 4). Study populations originated from diverse geographical regions, including 42 countries. Most studies were conducted in a single country, with the highest proportions from Europe (129/319, 40.4%) and North America (123/319, 38.6%), followed by Asia (40/319, 12.5%), South America (8/319, 2.5%), Africa (5/319, 1.6%), and Oceania (2/319, 0.6%). The remaining 12 (3.8%) studies were multicountry. Most studies targeted adolescents (aged 10-18 years; 130/319, 40.7%) and young adults (aged 18-26 years; 93/319, 29.1%), with 9 (2.8%) studies targeting both adolescents and young adults. Fewer studies focused on adult populations (aged ≥18 years; 68/319, 21.3%). The remaining 19 (6.0%) studies did not specify the age group of the target population. In terms of gender, 294 (92.2%) studies included both female and male populations, while 14 (4.4%) focused only on female populations and 6 (1.9%) focused only on male populations. Five studies (1.6%) included gender minority individuals, such as transgender and gender-diverse populations. Regarding sexual orientation, 314 (98.4%) studies did not specify the sexual orientation of the target population, and only 5 (1.6%) focused on sexual orientation minority populations. The studies used a variety of methodologies, including quantitative surveys (303/319, 95.0%), qualitative interviews (12/319, 3.8%), and mixed methods approaches (4/319, 1.2%).

Among the 319 included studies, 218 distinct measures of TFSVA were identified. Most of these measures were related to nonconsensual sexting and sextortion (130/218, 59.6%). Other areas addressed included IBSA or revenge pornography (30/218, 13.8%), online sexual harassment (27/218, 12.4%), online grooming or solicitation (13/218, 6.0%), and cyber sexual dating abuse (8/218, 3.7%). The remaining 10 (4.6%) measures assessed general TFSVA behaviors. In terms of measurement focus, 96 (44.0%) measures focused on exposure to violence, 12 (5.5%) on perpetration, and 108 (49.5%) measured both aspects. One (0.5%) measure focused on the bystander role, and 1 (0.5%) measured all aspects. In terms of outcomes measured, most studies assessed TFSVA behaviors (199/218, 91.3%), including outcomes related to the prevalence and experiences of TFSVA. Five (2.3%) measures assessed attitudes toward TFSVA, and 4 (1.8%) evaluated motivations for committing TFSVA. The remaining 10 (4.6%) measures assessed more than 1 outcome. Characteristics of the included studies are presented in Table 1 and TFSVA measures are summarized in Table 2.

**Table 1:** Characteristics of the included studies. This table summarizes the key methodological and contextual details of the included studies (N=319).

Characteristics		Value, n (%)
**Study population**		
	Europe	129 (40.4)
	North America	123 (38.6)
	Asia	40 (12.5)
	South America	8 (2.5)
	Africa	5 (1.6)
	Oceania	2 (0.6)
	Included more than 1 country	12 (3.8)
**Age group (years)**		
	Adolescents (aged 10-18)	130 (40.7)
	Young adults (aged 18-26)	93 (29.1)
	Adolescents and young adults	9 (2.8)
	Adults (aged 18 or above)	68 (21.3)
	Any age group	19 (6.0)
**Gender**		
	Both genders	294 (92.2)
	Female only	14 (4.4)
	Male only	6 (1.9)
	Gender minority (ie, transgender and gender-diverse)	5 (1.6)
**Sexual orientation**		
	Not specified	314 (98.4)
	Sexual orientation minority (eg, LGBTQ+^a^ or men who have sex with men)	5 (1.6)
**Study methodology**		
	Quantitative survey	303 (95.0)
	Qualitative interview	12 (3.8)
	Mixed methods study	4 (1.2)

^a^LGBTQ+: lesbian, gay, bisexual, transgender, queer (or questioning), and others not explicitly listed.

**Table 2:** TFSVA^a^ measures (n=218).

Characteristics		Value, n (%)
**Types of TFSVA**		
	Nonconsensual sexting	130 (59.6)
	Image-based sexual abuse	30 (13.8)
	Online sexual harassment	27 (12.4)
	Online grooming	13 (6.0)
	Cyber sexual dating abuse	8 (3.7)
	General TFSVA behaviors	10 (4.6)
**Measurement focus**		
	Exposure to violence	96 (44.0)
	Perpetration	12 (5.5)
	Exposure to violence and perpetration	108 (49.5)
	Bystander	1 (0.5)
	All aspects	1 (0.5)
**Outcome measures**		
	Behaviors	199 (91.3)
	Attitudes	5 (2.3)
	Motivations	4 (1.8)
	More than 1 outcome	10 (4.6)

^a^TFSVA: technology-facilitated sexual violence and abuse.

**Figure 2:**
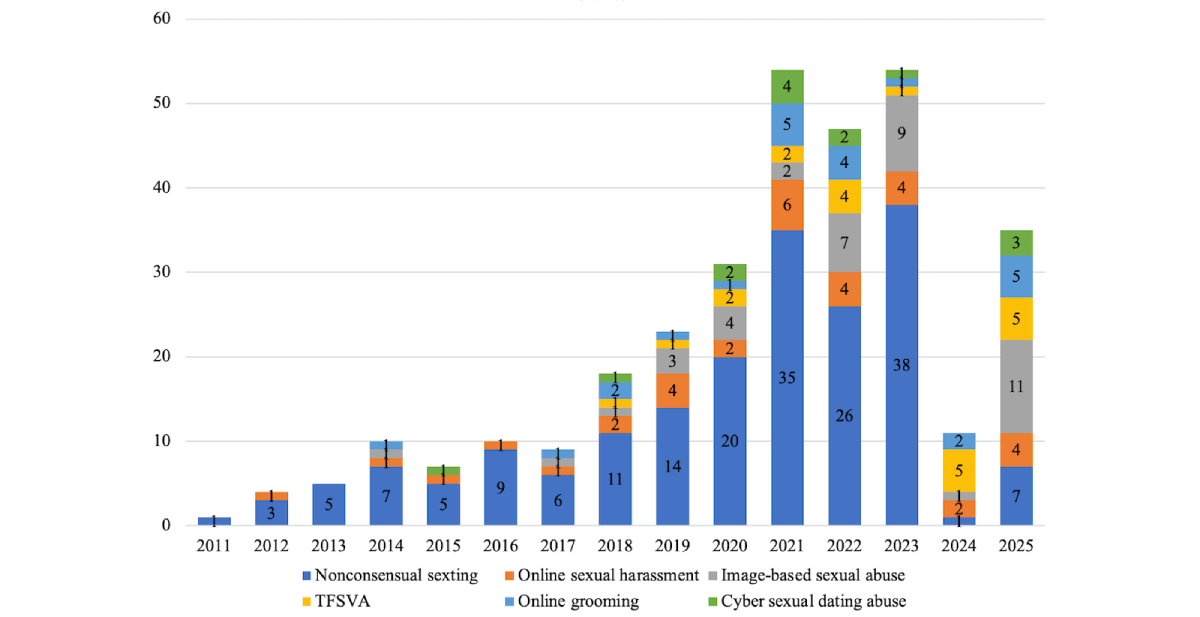
Time trends in publications on different types of technology-facilitated sexual violence and abuse (TFSVA), 2011-2025. This stacked bar chart displays the annual number of publications included in this review, categorized by primary focus.

Analysis of publication trends from 2011 to 2025 reveals the evolving focus of TFSVA measures in research. From 2011 to 2023, nonconsensual sexting emerged as the dominant type of TFSVA being measured, with numerous studies focusing on its prevalence, risks, and consequences, particularly among adolescents. However, since 2018, the field has expanded to include more diverse types of TFSVA, such as IBSA, online sexual harassment, and online grooming, reflecting the evolving nature of digital harms. The term TFSVA first appeared prominently in academic literature around 2018, marking a shift toward a more comprehensive understanding of technology-mediated sexual violence, and studies measuring TFSVA have increased significantly since then. By 2024, TFSVA had become the dominant focus of investigation, surpassing the narrower emphasis on nonconsensual sexting, with a broader spectrum of digital sexual violence and its multifaceted impacts being addressed. Data from 2025 indicate a continued increase in studies dedicated to these specific subtypes. The time trend of publications across different types of TFSVA is detailed in Figure 2.

**Figure 3:**
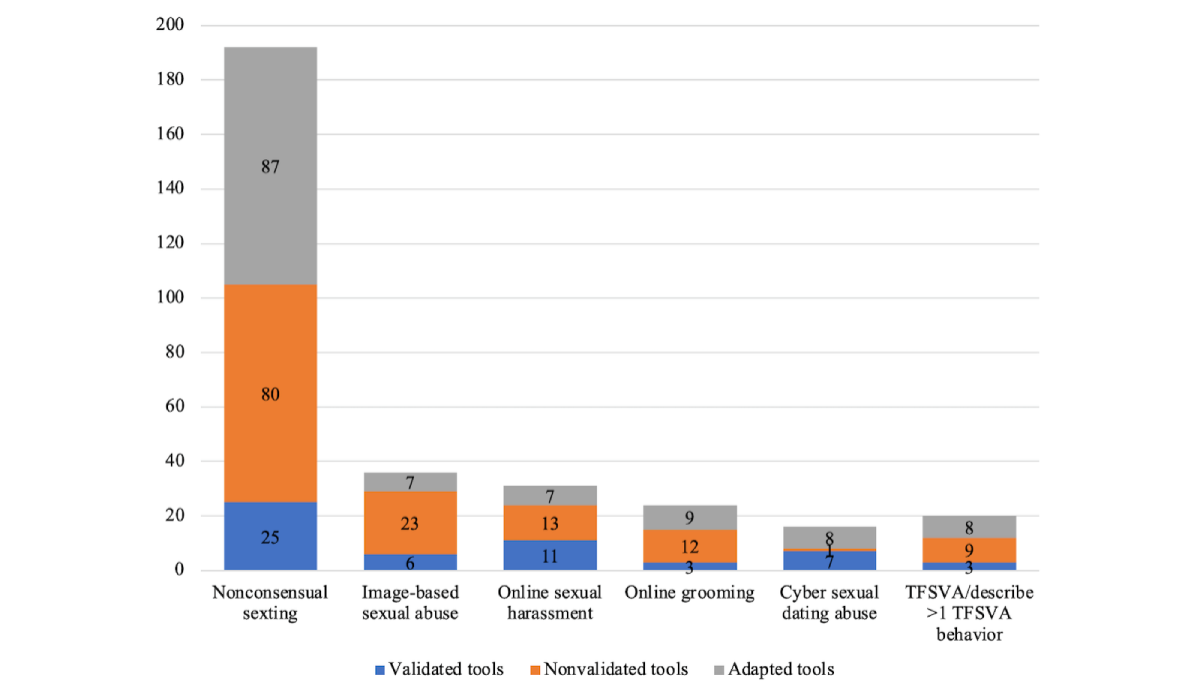
Validation status of measures by type of technology-facilitated sexual violence and abuse (TFSVA). This stacked bar chart displays the distribution of validated, nonvalidated, and adapted measures for each TFSVA type identified in the included studies.

Among all included studies, only 55 reported details on psychometric properties. A total of 138 studies used nonvalidated measures, and 126 studies adapted questionnaires from existing measures. The distribution of validated, nonvalidated, and adapted measures by type of TFSVA across all included studies is presented in Figure 3.

### Measures by Types of TFSVA

#### Nonconsensual Sexting

Nonconsensual sexting, defined as sending or receiving sexually explicit messages or images without one’s permission, was the most extensively studied form of TFSVA (130/218, 59.6%). In terms of age group, 56 out of 130 (43.1%) measures targeted adolescents, 39 out of 130 (30%) focused on young adults, and 32 out of 130 (24.6%) focused on adult populations; 2 out of 130 (1.5%) measures targeted both adolescents and young adults, and the remaining 1 out of 130 (0.8%) included all age groups. Three nonconsensual sexting measures specifically targeted female participants, and none of the measures specified the sexual orientation of the target population.

Nearly all studies related to nonconsensual sexting (n=127) focused on assessing the behavioral domain, with questions primarily addressing the frequency and targets of sexting. Six studies measured attitudes toward nonconsensual sexting, exploring general perceptions and perceived risks associated with the practice. Additionally, 7 studies investigated the motivations behind such behavior, examining reasons for engagement, including sexual purposes, emotional influences, body-image reinforcement, and relationship enhancement.

In terms of psychometric properties, 25 measures were validated, of which 17 reported validity analyses, such as exploratory factor analysis or confirmatory factor analysis. All validated measures reported reliability, including internal consistency (eg, Cronbach α or McDonald ω). Among the validated measures, the Sexting Motivation Scale [24], the Sexting Behavior Scale [25], the Sexting Questionnaire [26], and the Sexting Attitude Scale [27] were the most commonly used. All of these demonstrated strong validity (ie, satisfactory exploratory factor analysis or confirmatory factor analysis) and reliability (ie, Cronbach α >0.8). However, these tools were predominantly validated among adolescents and young adults (18/25, 72%), with limited application to adults or diverse cultural groups.

#### Online Sexual Harassment

Online sexual harassment refers to any unwelcome or unwanted sexual behavior, advances, or communication that occurs through digital platforms, including social media, messaging apps, email, or online gaming environments [28]. This form of harassment encompasses a broad range of behaviors, such as sexualized cyberbullying, nonconsensual sharing of intimate content, sexually explicit comments, cyberflashing, and sexual coercion facilitated by digital means. Among the included studies (n=218), 27 (12.4%) measured online sexual harassment. In terms of age group, 11 out of 27 (41%) targeted adolescents, 8 out of 27 (30%) targeted young adults, 6 out of 27 (22%) targeted the adult population, and the remaining 2 (7%) included all age groups; 6 out of 27 (22%) measures were distributed to the female population only, and 1 targeted males only. One measure specifically focused on lesbian, gay, bisexual, transgender, queer (or questioning), and others not explicitly listed (LGBTQ+) individuals and examined sexuality-based cyberbullying [29].

Almost all measures included behavioral outcomes (26/27), with 1 study also measuring the motivation outcome. One study explored the attitude outcome from a bystander perspective; 19 out of 27 (70%) measures focused on exposure to violence, 3 (11%) on perpetration, and the remaining 5 (19%) on both aspects. Most questions in these studies inquired about personal experiences of online sexual harassment, such as receiving offensive sexist messages, encountering negative comments about one’s gender or sexual orientation, or being subjected to inappropriate sexual remarks about one’s body on social media. One measure with a motivation outcome asked about reasons for online sexual harassment.

As many as 11 (41%) measures demonstrated satisfactory validity and reliability. The Online Sexual Victimization Scale was the most commonly used measure [26].

#### Image-Based Sexual Abuse

IBSA, such as the nonconsensual sharing of intimate images, was assessed in 30 out of 218 (13.8%) studies; 5 out of these 30 (17%) studies targeted adolescents, 13 (43%) targeted young adults, and 6 (20%) targeted adults; 4 (13%) studies included participants across all age groups, and the remaining 2 (7%) did not specify the target population. One study focused on the male population only, while the others did not specify gender or sexual orientation.

Most measures focused on behavioral outcomes (n=25), while 4 studies assessed attitude outcomes and 1 included motivation outcomes. Among the 30 studies, 14 (47%) focused on IBSA exposure, 3 (10%) on perpetration, 12 (40%) included both exposure to violence and perpetration, 1 (3%) focused on a bystander perspective, and 1 (3%) included all perspectives. The measurement questions explored experiences related to sending, receiving, or requesting intimate, suggestive, or provocative images or videos (eg, “I have sent/received/requested intimate/suggestive/provocative images or videos”), reasons and motivations for sharing such content (eg, “I have sent dick pics hoping to receive sexy pictures in return”), and attitudes toward IBSA behaviors (eg, “If a man sends a nude or sexual image to a partner, he can’t expect it will remain private”).

A total of 6 (20%) measures were validated, demonstrating high validity and reliability. Among these, the Sexual Image-Based Abuse Myth Acceptance Scale was the most widely adapted and utilized [30].

#### Online Grooming

Online grooming refers to the process by which an individual, typically an adult, establishes an emotional connection with a child or adolescent through digital platforms, such as social media or messaging apps, with the intent of exploiting them for sexual purposes [31]. Of the 218 measures, 13 (6.0%) were designed to examine online grooming. Of these 13, 7 (54%) measures targeted adolescents, 2 (15%) targeted adults, 1 (8%) targeted young adults, and the remaining 3 (23%) focused on both adolescents and young adults. No studies specifically targeted sexual and gender minorities.

All studies focused on behavioral outcomes, with 1 also exploring motivations underlying such behavior. Of the 13 studies, 11 (85%) assessed online grooming exposure, while the remaining 2 (15%) examined both online grooming exposure and perpetration. Most measurement questions centered on the frequency and experiences of online grooming (eg, “In the past 12 months, how often has an adult asked me for pictures or videos of myself with sexual content?”, “Someone has made sexual jokes or comments about me”).

In these studies, 3 (23%) scales were validated with high reliability and validity, among which the Questionnaire for Online Sexual Solicitation and Interactions With Adults was the most commonly used [32].

#### Cyber Sexual Dating Abuse

Cyber sexual dating abuse, encompassing behaviors such as monitoring, control, and harassment within romantic relationships, was addressed in 8 out of 218 (3.7%) studies. Of these 8, 5 (63%) focused on adolescents and 3 (38%) on adults. One study specifically targeted sexual and gender minority girls and feminine teens.

All studies focused on behavioral outcomes, with 5 (63%) specifically addressing exposure and 3 (38%) exploring both exposure and perpetration. Some questionnaire content overlapped with items in IBSA and sexual aggression in dating or intimate relationships (eg, “Sending and/or uploading photos, images, and/or videos with intimate or sexual content without permission,” “Pressured my partner to send sexual or naked photos of him or her to me”).

Of these 8 measures, 7 (88%) were validated, demonstrating high reliability and validity. Among these, the Cyber Dating Abuse Questionnaire was the most frequently adapted scale [33].

#### TFSVA as a Whole

Since 2018, the term TFSVA has gained prominence as a comprehensive descriptor for a wide range of technology-mediated sexual harms [28]. Of the 218 studies, 10 (4.6%) explored TFSVA as a broader category: 2 (20%) targeted adolescents, 7 (70%) targeted adults, and the remaining 1 (10%) had no age restrictions. No studies specifically targeted sexual and gender minorities.

Of the 10 measures, 9 (90%) focused on behavioral outcomes, and 1 (10%) on attitudes toward TFSVA. Among these studies, 6 (60%) examined TFSVA exposure, while 4 (40%) assessed both exposure and perpetration. The measures encompassed various forms of abuse, including IBSA, online sexual harassment, and gender- or sexuality-based harassment, often grouping these behaviors under the umbrella of TFSVA. Questionnaire items focused on experiences of TFSVA (eg, “Nude or seminude image taken without permission,” “Unwanted sexual experience with someone met online,” “Receiving sexuality- or sexual identity-based offensive and/or degrading messages, comments, or other content”).

Of the 10 measures, 3 (30%) were validated, with the 21-item Technology-Facilitated Sexual Violence Victimization Scale being widely adapted and utilized [34].

### Detailed Analysis of Validated Measures

To gain detailed insights into the most methodologically robust measures in the field, an in-depth analysis of the 55 validated measures was conducted. This subanalysis focused on their structural characteristics, including TFSVA-specific item length, measurement outcomes, target population, and psychometric properties.

The analysis focused on the TFSVA-specific item content within each validated measure. The number of items dedicated to assessing TFSVA constructs ranged from 1 to 63, with many measures embedding these items as a subscale within a broader questionnaire on general risky online behaviors or intimate partner violence. Validation was heavily concentrated in Western, high-income countries, notably the United States and Spain. Most measures targeted adolescent and young adult populations.

Psychometric reporting among validated measures followed a clear pattern. Internal consistency reliability (Cronbach alpha and McDonald ω) was commonly reported, with typical values exceeding 0.80. However, evidence for temporal stability (test-retest reliability) and construct validity (eg, convergent, discriminant, predictive) was reported less consistently and comprehensively. This indicates that the foundational reliability of TFSVA measures has been established, but the full spectrum of validation evidence is not yet universal. Detailed information on the validated measures and their use is synthesized in Multimedia Appendix 5.

### Content of Validated Measures

#### Content Analysis Using STM

**Figure 4:**
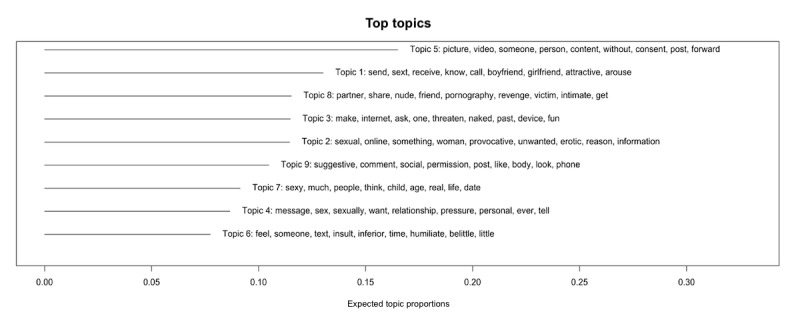
Summary of top topics identified by unsupervised structural topic modeling (STM). This figure presents the 9 most prevalent topics identified using unsupervised STM. For each topic, the bar represents overall prevalence, and the accompanying text lists the highest-probability keywords.

Topic interpretation primarily explains the topic words that describe each topic. STM identifies 9 topics based on high-frequency words. Figure 4 presents the top topics generated from unsupervised STM.

Topics were synthesized into 3 core dimensions representing the content of existing measures of TFSVA: (1) consent violation and coercive control; (2) status degradation and demeaning harassment; and (3) contextual and relational framing. Details of the measurement focus and representative topics for each theme are shown in Table 3.

**Table 3:** Core measurement dimensions identified from unsupervised structural topic modeling. This table presents the 3 primary dimensions identified through unsupervised structural topic modeling analysis of the included validated measures, listing their measurement focus and representative topics.

Dimensions	Measurement focus	Representative topics
Consent violation and coercive control	Measures acts that override consent, breach trust, and apply pressure or threats to dominate the survivor, representing an abuse of power.	3 (digital threats and control), 4 (sexual pressure online), and 5 (nonconsensual image distribution)
Status degradation and demeaning harassment	Measures acts designed to insult, humiliate, and lower the survivor’s social standing, which causes social and psychological harm.	2 (unwanted sexual advances and perceived context), 6 (psychological abuse), and 9 (body shaming)
Contextual and relational framing	Provides the situational setting by defining the specific relationship contexts or vulnerable populations that characterize the abuse.	1 (pressured sexting in relationships), 7 (sexual exploitation of minors), and 8 (intimate partner revenge porn and betrayal)

The first dimension, consent violation and coercive control, encompasses measures of acts that override consent, breach authorization, or apply pressure. It is constituted by topics 3, 4, and 5. For instance, topic 5, the most prevalent topic, captures the nonconsensual distribution of intimate images, anchoring a central TFSVA construct in consent violation. Topics 3 and 4 complement this by measuring coercive tactics, with topic 3 focusing on digital threats and topic 4 capturing direct sexual pressure.

The second dimension, status degradation and demeaning harassment, captures measures of acts intended to insult, humiliate, and lower the survivor’s social standing. Represented by topics 2, 6, and 9, it includes unwanted sexual advances that may carry attributions of blame (topic 2), psychological abuse and humiliation (topic 6), and body shaming in social media spaces (topic 9).

The final dimension, contextual and relational framing, defines the specific relational contexts and vulnerable populations that characterize TFSVA. It is represented by topics 1, 7, and 8. Topics 1 and 8 frame abuse within intimate relationships, measuring pressured sexting and revenge porn, respectively. Topic 7 captures online grooming and the sexual exploitation of minors.

**Figure 5:**
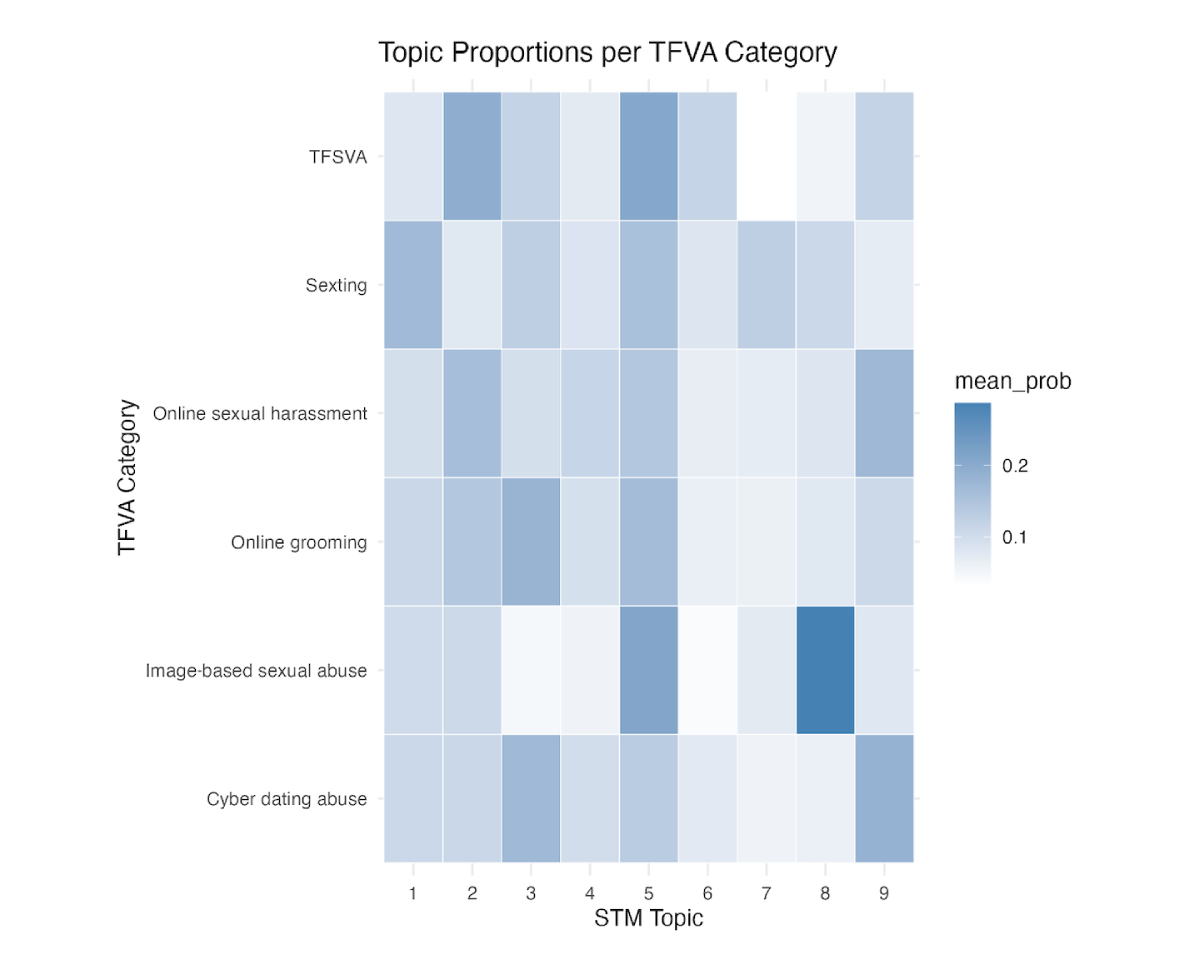
Topic prevalence by technology-facilitated sexual violence and abuse (TFSVA) type. This heatmap displays the prevalence of each STM topic (columns) across literature focused on specific TFSVA types (rows), highlighting which topics are central or peripheral to TFSVA measures.

Topic prevalence by types of TFSVA is shown in a heatmap (Figure 5). First, topic 5 (nonconsensual image distribution) demonstrates consistently high probability across all TFSVA types, suggesting it is a core element in TFSVA measures. Second, the relatively lower overall prevalence of topic 7 (online grooming and sexual exploitation of minors) may indicate that this form of abuse is underrepresented in general TFSVA instruments or is more often specialized in measures targeting child sexual exploitation. Third, the high specificity of certain topics—such as topic 8’s strong association with IBSA and the notable absence of topics 3 (digital threats) and 6 (psychological abuse) from the same type—indicates that IBSA is operationalized primarily around the violation of consent for visual content rather than coercive or psychological tactics.

#### Thematic Analysis

##### Theme 1: TFSVA Acts and Experiences

Results revealed that the conceptual foundation of TFSVA measures is the direct quantification of concrete acts and exposure experiences. This behavioral core is operationalized through a mirrored structure across instruments: perpetration tools inventory behaviors enacted, while victimization tools inventory exposure to those same behaviors. This is evidenced by the parallelism in items such as a perpetration subscale asking, “In the past year, have you asked someone to send naked pictures of them to you? When received, I’ve forwarded or shared suggestive or sexual text messages of other people” [35,36], and a victimization scale probing, “I’ve received suggestive or sexual text messages” [35].

Beyond this dichotomy, measures capture critical behavioral specifics that define the phenomenon in the modern context. This included the nature of content shared (eg, “suggestive or sexual text messages/videos/images”), the exploitation of specific digital platforms (eg, “social media like Facebook, YouTube, Twitter, Instagram/messaging and communication apps like WhatsApp/online forums and chatrooms/websites and blogs”), and the relational dynamics between parties involved (eg, “friends or peers/online friends/stranger/romantic partner). Furthermore, instruments employed frequency metrics (eg, “number of times in the past 6 months that the participant had sent/received a sexual message”) to quantify experiences [37]. A few questionnaires also included behavioral continuums ranging from online harassment to physical violence (eg, “threatened on a digital device to physically hurt me”) [38].

##### Theme 2: TFSVA-Related Attitudes and Beliefs

The analysis identified that a distinct subset of instruments moved beyond measuring acts to assess the underlying cognitive frameworks that perpetuate TFSVA. These tools were designed to capture the beliefs that justify, minimize, or shift responsibility for abusive behaviors, and they revealed a critical triangulation of perspectives across different populations. Perpetrator-focused instruments measured aggression-supporting cognitions that legitimize harmful acts, exemplified by items asserting that “Women should be flattered if a partner or ex-partner shows nude pics of her to some close friends” [30]. Conversely, survivor-focused tools often assessed internalized blame, capturing the harmful internalization of these same societal myths through items such as, “Someone who sends a nude or sexual image to their partner, should not be surprised and is responsible if the image ends up online” [30]. Furthermore, instruments gauged societal-level myths and misconceptions that form the bedrock of these attitudes, including normative assertions like “It’s only natural for a guy to brag to his mates by showing them a nude or sexual image of his partner” [30].

##### Theme 3: Motivational Factors in TFSVA

This theme examined the complex motivational factors underlying TFSVA, capturing the distinct methodological approaches used to ascertain reasons for perpetration. The analysis revealed that motivations were conceptualized along a continuum spanning internally generated, autonomous reasons and externally driven, coerced reasons, typically measured through direct self-report questions posed to perpetrators. Autonomous motivations encompassed internally driven drivers such as relationship enhancement (eg, “sometimes I sent sexts to increase intimacy in my dating relationship”), sexual arousal (eg, “I sent sexts to feel sexually aroused”), and self-validation (eg, “I sent sexts to test whether I am attractive enough/ to verify whether my body is okay”) [24]. In direct contrast, coerced motivations were characterized by external pressure or instrumental gain, encompassing factors such as blackmail (eg, “sent threatening messages on digital devices”) [38], compliance with external pressures (eg, “After you sent the sext, did anyone praise you/did you feel like you fit in better with your friends/peers?”) [39], and the pursuit of tangible benefits such as financial rewards (eg, “offered me money or other things in exchange”) [40]. A critical methodological divergence was also identified, whereby perpetrator motivation was alternatively assessed through the survivor’s interpretation of the event (eg, “perpetrator did this to humiliate me/to feel a sense of dominance and control”) [41].

##### Theme 4: The Tactical and Contextual Dynamics of TFSVA

Measures of TFSVA demonstrated a sophisticated focus on the specific perpetrator tactics and contextual dynamics that define TFSVA. Instruments consistently captured a core set of direct harassment behaviors, quantifying the nonconsensual sharing of intimate images and the use of persistent online sexual threats through items probing experiences of “unwanted sexual communication” and “IBSA.”

Beyond these direct acts, instruments increasingly measured more nuanced and calculated strategies. This included deception and impersonation, assessed through items asking respondents whether they had been contacted by “fake profiles” or had experienced “false rumors about their sexual behaviors being spread online” [42]. A critical tactical subset involved overt coercion, prominently featured in the operationalization of sextortion. This was measured through items detailing how perpetrators manipulate survivors through threats, such as “Has anyone ever threatened to share your private images unless you sent money or more photos?,” which captures the dual mechanisms of financial coercion and sextortion [40].

##### Theme 5: Survivor Impact and Harm

Measures of impact revealed a primary focus on the psychological and relational consequences for survivors of TFSVA. Existing instruments predominantly assessed 2 core internalized harms: internalized blame and trauma-related emotional effects. The first focused on items that probe self-directed blame and responsibility, such as survivors endorsing statements such as “senders should expect images to be shared” [41]. The second captured the profound emotional fallout, quantifying experiences of humiliation, violated trust, and social anxiety through items like “victims find it difficult to trust others/often experience feelings of humiliation/being harassed or annoyed.” [41,43].

Beyond internal states, a significant portion of questions addressed broader relational outcomes, often framed by a tension between perceived positive effects (eg, intimacy-building within a consensual context) and the more frequently documented negative consequences of regret, trauma, and the reinforcement of power imbalances (eg, “Did you experience regret immediately after sending the sext?/I sent sexts because I am forced by someone”) [39,44]. Crucially, the nonconsensual nature of the acts was central to the conceptualization of harm. The profound impact was explicitly linked to the experience of violation, whether through the unauthorized sharing of intimate content, the coercion of sextortion, or the fundamental breach of privacy (eg, “disseminating or uploading to the Internet photos or videos of you with erotic or sexual content without your consent”) [45].

#### Integrated Synthesis

**Figure 6:**
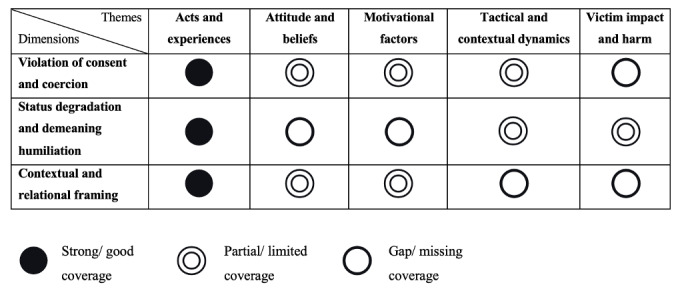
Synthesis of content coverage from structural topic modeling and thematic analysis. The table compares the 3 core measurement dimensions identified by structural topic modeling with the 5 themes derived from thematic analysis, indicating their coverage in the current evidence as strong, limited, or missing.

To integrate the findings from STM and thematic analysis, an integrated evidence and gap map was developed and is presented in Figure 6. This conceptual synthesis does not propose a direct correlation but serves as an evaluative framework. It positions the 3 core measurement dimensions identified through STM against the 5 themes derived from thematic analysis. For each dimension, judgment was made regarding its coverage of each thematic construct. The resulting matrix reveals a structural pattern in which robust measurement of behavior differs significantly from deficits in assessing attitudes, motivations, tactical dynamics, and harm-related components.

## Discussion

### Summary of Evidence

This scoping review provides a novel diagnostic analysis of the TFSVA measurement field. Through the integration of STM and thematic analysis of 319 studies encompassing 218 distinct measures, we reveal a fundamental structural misalignment in the field. Current measures are heavily concentrated on cataloging specific behavioral acts while offering only limited or partial coverage of underlying attitudes, motivations, harm-based dimensions, and systemic contexts. This fragmentation helps explain why the field continues to struggle with conceptual and methodological challenges in capturing the multifaceted nature of TFSVA.

### Interpretation and Comparison With Existing Literature

#### Scope and Definition of TFSVA

This review confirms the definitional fragmentation of TFSVA noted in prior literature. Previous studies have observed that TFSVA encompasses a diverse range of behaviors studied in isolation using inconsistent terminology [28]. Henry and Powell’s [28] foundational work identified a critical lack of empirical research on TFSVA against adults and noted that only a few existing studies provide reliable data on the nature, scope, and impacts of TFSVA. Nearly a decade later, our synthesis provides empirical confirmation of this fragmentation. The field has produced an abundance of measures, yet they remain focused on specific behavioral subdomains with minimal conceptual integration.

Three key findings from our analysis directly inform the need for an updated definition. First, the narrow behavioral focus of existing measures reveals that current conceptualizations fail to capture the full spectrum of TFSVA. Second, our analysis identified emerging forms of TFSVA, such as AI-generated deepfakes, that are absent from existing measures. Third, our findings show that TFSVA operates within broader structural contexts that shape both the nature of abuse and its consequences.

Grounded in this evidence, we propose an updated definition that also engages with distinctions raised in restorative and transformative justice scholarship [46]. Building on this foundation, we define TFSVA as any form of sexual violence, abuse, coercion, or exploitation that is initiated, facilitated, intensified, or sustained through digital technologies. Such acts may result in psychological, reputational, social, economic, physical, or sexual harm, though the presence or absence of harm is not definitional to the act itself.

This formulation separates the act from its potential consequences. It acknowledges that (1) individuals may experience TFSVA without immediate measurable harm yet still warrant recognition and support; (2) the same act may produce different harms across individuals and contexts; and (3) harm may manifest differently across cultural and geographical settings [46].

The definition encompasses specific acts documented in our review, including IBSA, AI-generated sexual exploitation, cyberstalking leading to physical violence, and algorithmically amplified harassment. It remains adaptable to emerging forms not yet captured in existing measures. Crucially, it recognizes that TFSVA operates within systems of gendered, racialized, and intersectional oppression. These dimensions, which often transcend digital or physical boundaries and jurisdictional borders, are currently absent from existing measures.

Additionally, the definition embeds recognition of systemic oppression and acknowledges that technology is not merely a neutral tool. For instance, algorithms can amplify harassment, platform design may facilitate sexual abuse, and networked technologies enable harm across jurisdictional boundaries [47]. These dimensions follow directly from a definition centered on technological and structural contexts.

#### Temporal Trends

The increase in TFSVA publications observed in our temporal analysis suggests growing academic and societal recognition. A notable trend is the decline in studies on specific forms, such as sexting and IBSA, from 2011 to 2017. The use of the holistic term “TFSVA” began to rise sharply from 2018, suggesting a conceptual shift in which previously distinct behaviors are being consolidated under a broader umbrella [3]. Additionally, the decline in specific terms may reflect a move away from fragmented terminology toward more comprehensive approaches that capture the spectrum of technology-facilitated sexual harms. Alternatively, it may indicate that earlier research on sexting, IBSA, online sexual harassment, and online grooming reached a saturation point, prompting scholars to explore broader themes or emerging forms of TFSVA. Further investigation is needed to determine whether this trend reflects evolving academic priorities or a response to new technological developments that blur the boundaries between specific subtypes of TFSVA.

#### Gender, Geographic, and Cultural Considerations

A core finding of this review is the gap in demographic representation within TFSVA measures. Henry and Powell [28] observed that TFSVA may be predominantly gender-, sexuality-, and age-based, with young women being overrepresented as survivors. However, our analysis reveals that the field lacks the measures needed to adequately investigate these patterns.

The gendered nature of TFSVA is well documented, with women and sexual minorities disproportionately affected [48]. Emerging evidence also suggests that men experience TFSVA, albeit in different forms and with varying impacts [49,50]. A recent study assessing the content validity of a TFSVA measure further highlighted significant variations in how individuals of different genders and sexual orientations interpret and perceive TFSVA [51].

Yet, our synthesis reveals 2 critical gaps that prevent the field from advancing this understanding. First, existing measures may lack content sensitivity to diverse identities. Items often use generic language that may not capture experiences specific to sexual and gender minorities. Second, even if measures could capture diverse experiences, they have rarely been validated with diverse populations. Of the 319 studies, 314 (98.4%) did not specify participants’ sexual orientation, and only 5 (1.6%) targeted gender minority populations. The implications of these gaps for measure design and validation are addressed in the subsequent discussion.

Moreover, the geographic focus of TFSVA research is heavily skewed. For instance, our results indicate that 260 (81.5%) of the 319 included studies were conducted in Western, high-income countries, while studies from Asian, African, and low-income contexts remain scarce. This imbalance is critical, as cultural norms and contextual factors, such as varying digital literacy, internet access, and legal frameworks, can significantly influence how TFSVA is perceived and experienced [3]. Consequently, existing measures may lack validity in these regions, highlighting the need to develop culturally adapted measures that account for specific social, legal, and technological environments.

#### Gaps and Recommendations

The thematic analysis of questionnaire content confirmed that existing measures capture behavioral data and certain attitudinal and motivational factors, but lack depth. Our integrated synthesis places these content gaps within a structural framework, revealing substantial limitations in addressing critical dimensions, notably evolving technological threats, intersectional vulnerabilities, and institutional factors.

Emerging technologies present challenges for TFSVA measures. Our analysis showed that emerging forms of TFSVA, such as AI, deepfakes, zoom-bombing or digital intrusion, and abuse via smart devices, were not covered in the measurement content. These gaps render existing questionnaires outdated and create a persistent development lag, as the evolution of abuse tactics outpaces instrument updates [51]. To remain relevant, future questionnaires should be dynamically updated to integrate assessments of emerging tactics, such as GPS stalking and AI-facilitated abuse.

Results from our analysis reveal limitations in capturing intersectional experiences at 2 levels. First, in terms of measure design, the content of existing measures lacks sensitivity to how TFSVA manifests across individuals of different genders and sexual orientations. Items often use generic language (eg, “Have you received unwanted sexual images?”) that does not account for specific experiences among sexual and gender minority populations. This limits the validity of these measures for diverse groups. Second, in terms of study application, our descriptive synthesis shows that most included studies did not specify participants’ sexual orientation. This indicates that studies rarely conduct and report the intersectional analyses needed to understand disparities.

Existing literature on other sexual violence measures offers concrete guidance for improving inclusivity. For example, the Daily Heterosexist Experiences Questionnaire includes items that specifically assess discrimination and harassment tied to sexual minority identity, such as “People assuming you are heterosexual because you have children,” moving beyond generic violence exposure questions [52]. Similarly, measures of intimate partner violence for sexual and gender minority populations adapt items from the Revised Conflict Tactics Scale to capture experiences unique to these populations, including identity-based coercion and outing as a tactic of abuse [53]. These examples demonstrate that inclusive measures require consultation with diverse communities during item development to ensure content validity [52]. A second strategy is the use of language that reflects the lived experiences of specific groups rather than assuming universal applicability [53]. A third is validating measures separately across different populations to establish measurement equivalence [53]. Incorporating these strategies into TFSVA measure development is crucial for capturing nuanced harms, such as gendered and sexuality-based abuse [5]. Future measure development should prioritize inclusive language and item wording, community engagement in the design process, and rigorous validation across diverse gender and sexual orientation groups.

A related and critical gap is the lack of cross-cultural validity. Our findings show that most validated measures were developed in Western, high-income countries. To move forward, the adaptation of TFSVA measures should follow a rigorous framework to ensure validity across different contexts [54]. This involves (1) ensuring conceptual equivalence through co-creation with local experts and cognitive testing; (2) conducting statistical measurement invariance testing (eg, multigroup confirmatory factor analysis); and (3) establishing criterion validity. This structured process moves beyond simple translation to develop measures that are valid for cross-cultural research and intervention.

Findings from the thematic analysis of measurement content indicate that current TFSVA-specific measures critically underassess structural and institutional factors, such as interactions with legal systems and digital platforms. There is a notable absence of items within TFSVA measures assessing key mediators of survivor outcomes, such as a survivor’s trust in, or experience with, platform reporting systems, legal authorities, or support services. It is important to note that this finding is limited to the content of TFSVA-specific measures. Our review did not capture whether included studies administered additional, standalone measures assessing institutional trust, platform responsiveness, or help-seeking behaviors alongside their TFSVA measures. Therefore, although these constructs are absent from TFSVA-specific measures, we cannot draw conclusions regarding their overall availability or use elsewhere in the literature. Nevertheless, the absence of these constructs from TFSVA-specific measures has important implications. A growing body of evidence demonstrates that institutional responses profoundly influence survivor outcomes [55]. Research on situational betrayal theory has shown that platform reporting processes can themselves become sources of secondary trauma when they are hostile, opaque, or ineffective [56]. Institutional responses also shape bystander behaviors and help-seeking pathways [57]. For instance, bystanders’ willingness to intervene is influenced by their perceptions of safety, responsibility, and the anticipated consequences of action, which are shaped by broader institutional and social norms [57]. Similarly, survivors’ decisions to seek support are mediated by their trust in institutional responses and fear of further harm during or following disclosure [57]. Consequently, when TFSVA measures are used in isolation, they can quantify the abusive act but cannot assess the institutional and structural context in which harm is experienced and mediated. To build an evidence base for effective interventions, future TFSVA measure development should consider integrating items that assess constructs such as institutional trust, platform responsiveness, and multilevel help-seeking behaviors.

Our analysis of TFSVA-specific measures revealed limited attention to psychological and relational impacts, such as long-term trauma, reputational harm, or intimate partner dynamics. Results from STM and thematic analysis indicate that when impacts are included, they tend to frame consequences as isolated emotional states rather than examining how they interact with, or amplify, other mental health challenges [58]. However, this finding is limited to the content of TFSVA measures, as we did not capture whether included studies administered separate validated instruments assessing psychological outcomes alongside their TFSVA-specific measures. Future research should examine how TFSVA measures are used in conjunction with established mental health measures. Additionally, TFSVA measure development should consider integrating items that capture long-term and relational impacts to provide a more comprehensive understanding of survivors’, perpetrators’, and bystanders’ outcomes.

### Limitations

This scoping review has several limitations. First, by design, it maps the available evidence but does not appraise study quality or synthesize findings, which precludes definitive conclusions regarding measurement robustness. Second, STM was applied to short questionnaire items. The required text preprocessing may have fragmented the original wording; however, topics were interpreted against the original texts to ensure validity. Third, the inclusion of only English and Chinese publications may have introduced language bias, potentially omitting relevant studies. Fourth, as TFSVA is an evolving field, this review may not capture all emerging forms of the phenomenon. Fifth, our analysis focused specifically on TFSVA measures rather than all measures used within the included studies. Future research could examine how TFSVA measures are used alongside other measures assessing related constructs, such as institutional trust or mental health outcomes.

### Conclusions

This scoping review provides the first comprehensive synthesis of existing TFSVA measures through the innovative integration of STM and thematic analysis. By examining 319 studies and 218 distinct measures, we move beyond cataloging to systematically identify the core dimensions reflected in existing measures, such as consent violations, coercive control, and contextual-relational framing. The review also reveals a field that is structurally misaligned with the realities of TFSVA: (1) a lack of measures addressing emerging AI-facilitated tactics; (2) the systematic omission of intersectional factors, such as gender and sexuality; (3) insufficient attention to structural and institutional mediators, such as platform responsiveness; and (4) superficial assessments of psychological and relational impacts, including long-term trauma. Importantly, these findings reflect gaps within TFSVA-specific measures rather than the broader literature, as we did not capture whether studies administered separate measures assessing these constructs alongside their TFSVA measures.

This approach quantifies the field’s conceptual fragmentation and pinpoints empirical blind spots. The primary contribution is an evidence-based analysis that redirects measures from narrow, tactic-focused content toward capturing more diverse contextual dimensions. To address the identified gaps, future efforts must prioritize: (1) developing measures with inclusive, community-informed content that reflects diverse experiences across gender, sexuality, and cultural contexts; (2) validating measures across diverse populations to establish measurement equivalence; and (3) integrating items that assess institutional and structural factors shaping TFSVA outcomes. This shift is essential for generating the comprehensive evidence needed to inform responsive policy, platform governance, and social support services in the rapidly evolving digital age.

## Data Availability

This study analyzed secondary data sourced from the existing published literature. The complete set of references is provided in Multimedia Appendix 4.

## Appendix

Multimedia Appendix 1 Keywords and search strategies.

Multimedia Appendix 2 PRISMA-ScR (Preferred Reporting Items for Systematic Reviews and Meta-Analyses Extension for Scoping Reviews) checklist.

Multimedia Appendix 3 PRISMA-S (PRISMA Statement for Reporting Literature Searches in Systematic Reviews) checklist. PRISMA: Preferred Reporting Items for Systematic Reviews and Meta-Analyses.

Multimedia Appendix 4 All included references.

Multimedia Appendix 5 Summary of validated measures.

## Abbreviations

AI: artificial intelligence
IBSA: image-based sexual abuse
LGBTQ+: lesbian, gay, bisexual, transgender, queer (or questioning), and others not explicitly listed
PAGER: Patterns, Advances, Gaps, Evidence for practice, and Research Recommendations
PCC: Population-Concept-Context
PRISMA-S: PRISMA Statement for Reporting Literature Searches in Systematic Reviews
PRISMA-ScR: Preferred Reporting Items for Systematic Reviews and Meta-Analyses Extension for Scoping Reviews
STM: structural topic modeling
TFSVA: technology-facilitated sexual violence and abuse
