# Supplementary material for: Measuring Technology-Facilitated Sexual Violence and Abuse: Scoping Review of Existing Measures
Source: J Med Internet Res. 2026 Apr 14;28:e90068. doi: 10.2196/90068 (PMC13125977; doi:10.2196/90068)
Supplement: Multimedia Appendix 1 [file jmir_v28i1e90068_app1.docx]

**Multimedia Appendix 1: Keywords and search strategies**

**Searching terms:**

| Technology & sexual abuse | - Cyberbullying - Cyberstalking - Cybermob - Deepfake - Doxxing - Image-based sexual abuse - Non-image based sexual abuse - Online grooming - Online initiated physical sexual violence - Online impersonation - Online sexual harassment - Online sexual violence/ abuse - Revenge porn - Sexting/ sextortion - Shallowfake - Technology-facilitated sexual violence/ abuse/ violence and abuse |
| --- | --- |
| Sexual abuse | - Commercial sexual exploitation - Child sexual abuse material - Child pornography - Intimate partner/ spousal/ domestic violence - Rape - Sexual abuse - Sexual assault - Sexual exploitation - Sexual harassment - Sexual offense - Sexual violence - Sexualized material |
| Technology | - Blued - Dating app - Digital - Grindr - Image - Image-based - Livestream - Online - Social media - Technology - Technology-facilitated - Tinder - Technology-assisted - Video - Video game - Web - Web-based - WhatsApp - WeChat |
| Measurement | - Measurement - Measure - Scale - Tool - Instrument - Index - Validation - Scale development - Questionnaire - Survey |

**Search strategy:**

***PubMed* – original search on 31 December, 2024**

| Set | Terms |
| --- | --- |
| #1 | Cyberbullying[All Fields] OR cyberstalking[All Fields] OR cybermob[All Fields] OR deepfake[All Fields] OR doxxing[All Fields] OR "image-based sexual abuse"[All Fields] OR "non-image-based sexual abuse"[All Fields] OR "online grooming"[All Fields] OR "online initiated physical sexual violence"[All Fields] OR "online impersonation"[All Fields] OR "online sexual harassment"[All Fields] OR "online sexual violence"[All Fields] OR "online sexual abuse"[All Fields] OR "online sexual exploitation"[All Fields] OR "revenge porn"[All Fields] OR sexting[All Fields] OR sextortion[All Fields] OR shallowfake[All Fields] OR "technology-facilitated sexual violence"[All Fields] |
| #2 | Rape[All Fields] OR "sexual abuse"[All Fields] OR "sexual assault"[All Fields] OR "sexual exploitation"[All Fields] OR "sexual harassment"[All Fields] OR "sexual offense"[All Fields] OR "sexual violence"[All Fields] OR "intimate partner violence"[All Fields] OR "spousal violence"[All Fields] OR "domestic violence"[All Fields] OR "sexualized material"[All Fields] OR "commercial sexual exploitation"[All Fields] OR "child sexual abuse material"[All Fields] OR "child pornography"[All Fields] |
| #3 | Technology[All Fields] OR online[All Fields] OR web[All Fields] OR "technology-facilitated"[All Fields] OR "web-based"[All Fields] OR image[All Fields] OR "image-based"[All Fields] OR video[All Fields] OR WhatsApp[All Fields] OR WeChat[All Fields] OR Tinder[All Fields] OR Blued[All Fields] OR Grindr[All Fields] OR "social media"[All Fields] OR "video game"[All Fields] OR "dating app"[All Fields] OR livestream[All Fields] OR digital[All Fields] |
| #4 | "Measure"[Mesh] OR "Scale"[Mesh] OR "Questionnaire"[Mesh] OR "Survey"[Mesh] OR validation[All Fields] OR "scale development"[All Fields] OR index[All Fields] |
| #5 | (#1 OR (#2 AND #3)) AND #4 |
| #6 | #5 AND ((comparativestudy[Filter] OR interview[Filter] OR observationalstudy[Filter] OR randomizedcontrolledtrial[Filter] OR validationstudy[Filter]) |
| #7 | Publications from database inception to 31 December 2024 |

***PubMed* – Updated search on 13 January 2026**

- Date range from 1 January 2025 to 13 January 2026
- The search strategy and logic (line #1 to #6) remained identical to the original search. The data filter in line #7 was updated to cover the new period (#6 AND ("2025/01/01"[Date - Publication] : "2026/01/13"[Date - Publication]).
- 62 new records retrieved

***Embase* – original search on 31 December 2024**

| Set | Terms |
| --- | --- |
| #1 | ('Cyberbullying' or 'cyberstalking' or 'cybermob' or 'deepfake' or 'doxxing' or 'image-based sexual abuse' OR' non-image-based sexual abuse' or 'online grooming' or 'online initiated physical sexual violence' or 'online impersonation' or 'online sexual harassment' or 'online sexual violence' or 'online sexual abuse' or 'online sexual exploitation' or 'revenge porn' or 'sexting' or 'sextortion' or 'shallowfake' or 'technology-facilitated sexual violence').mp. [mp=title, abstract, heading word, drug trade name, original title, device manufacturer, drug manufacturer, device trade name, keyword heading word, floating subheading word, candidate term word] |
| #2 | ('rape' or 'sexual abuse' or 'sexual assault' or 'sexual exploitation' or 'sexual harassment' or 'sexual offense' or 'sexual violence' or 'intimate partner violence' or 'spousal violence' or 'domestic violence' or 'sexualized material' or 'commercial sexual exploitation' or 'child sexual abuse material' or 'child pornography').mp. [mp=title, abstract, heading word, drug trade name, original title, device manufacturer, drug manufacturer, device trade name, keyword heading word, floating subheading word, candidate term word] |
| #3 | ('technology' or 'online' or 'web' or 'technology-facilitated' or 'web-based' or 'image' or 'image-based' or 'video' or 'WhatsApp' or 'WeChat' or 'Tinder' or 'Blued' or 'Grindr' or 'social media' or 'video game' or 'livestream' or 'digital').mp. [mp=title, abstract, heading word, drug trade name, original title, device manufacturer, drug manufacturer, device trade name, keyword heading word, floating subheading word, candidate term word] |
| #4 | (Measur* or scal* or tool* or instrument* or question* or survey* or validat* or scale development or index).mp. [mp=title, abstract, heading word, drug trade name, original title, device manufacturer, drug manufacturer, device trade name, keyword heading word, floating subheading word, candidate term word] |
| #5 | (#1 OR (#2 AND #3)) AND #4 |
| #6 | Limit #5 to (chinese or english) AND article) |
| #7 | Publications from database inception to 31 December 2024 |

***Embase* – Updated search on 13 January 2026**

- Date range from 1 January 2025 to 13 January 2026
- The search strategy and logic (line #1 to #6) remained identical to the original search. The data filter in line #7 was updated to cover the new period (#6 AND yr="2025 -Current").
- 850 new records retrieved

***CINAHL* – original search on 31 December 2024**

| Set | Terms |
| --- | --- |
| #1 | (“cyberbully*” OR “ cyberstalk*” OR “cybermob” OR “doxing” OR “image-based sexual abuse” OR “non-image-based sexual abuse” OR “online initiated physical sexual violence” OR “technology-facilitated sexual violence” OR “technology-facilitated sexual abuse” OR “image-based sexual abuse” OR “online sexual violence” OR “online sexual harassment” OR “online sexual exploitation” OR “online sexual abuse” OR sext* OR “sextortion” OR “online groom*” OR “revenge porn” OR “shallowfake”) [all fields] |
| #2 | (“sexual abuse” OR “sexual violence” OR “sexual harassment” OR “sexual exploitation” OR “rape” OR “sexual assault” OR “sexual offense” OR “intimate partner violence” OR “spousal violence” OR “domestic violence” OR “sexualized material” OR “commercial sexual exploitation” OR “child sexual abuse material” OR “child pornography”) [all fields] |
| #3 | (“technology” OR “online” OR “web” OR ‘”echnology-facilitated” OR “web-based” OR “image” OR “image-based” OR “video” OR “WhatsApp” OR “WeChat” OR “Tinder” OR “Blued” OR “Grindr” OR “social media” OR “video game” OR “app” OR “livestream” OR “digital”) [all fields] |
| #4 | (Measur* OR scal* OR tool* OR instrument* OR question* OR survey* OR validat* OR scale development OR index) [all fields] |
| #5 | (#1 OR (#2 AND #3)) AND #4 |
| #6 | Limited to Chinese and English language, from database inception to current, journal article, questionnaire/scale, randomized controlled trial, research instrument, interview |

***CINAHL –* updated search on 13 January 2026**

- Date range from 1 January 2025 to 13 January 2026
- The search strategy and logic (line #1 to #6) remained identical to the original search. The data filter in line #7 was updated to cover the new period from 2025 to 2026.
- 109 new records retrieved

***Scopus* – original search on 31 December 2024**

| Set | Terms |
| --- | --- |
| #1 | TITLE-ABS-KEY (“technology-facilitated sexual violence” OR “technology-facilitated sexual abuse” OR “technology-facilitated gender-based violence”) OR TITLE-ABS-KEY (“cyberstalking” OR “cyberbullying” OR “cybermob”) OR TITLE-ABS-KEY (“online sexual violence” OR “online sexual harassment” OR “online sexual exploitation”) OR TITLE-ABS-KEY (“image-based sexual abuse” OR “sexting” OR “sextortion” OR “online grooming” OR “doxing” OR “revenge porn” OR “deepfake” OR “shallowfake”) |
| #2 | ALL (sexual abuse OR sexual violence OR sexual harassment OR sexual exploitation OR sexual assault OR sexual offense OR rape OR commercial sexual exploitation) OR ALL (intimate partner violence OR spousal violence OR domestic violence) OR ALL (sexualized material OR child sexual abuse material OR child pornography OR sexualized material) |
| #3 | ALL (technology OR online OR web OR technology-facilitated OR web-based OR digital) OR ALL (app OR WhatsApp OR WeChat OR Tinder OR Blued OR Grindr OR dating app OR social media OR video game OR livestream) OR ALL (image OR video) |
| #4 | ALL (measure* OR scale* OR tool* OR instrument*) OR ALL (questionnaire* OR survey* OR validat* OR scale development OR index) |
| #5 | (#1 OR (#2 AND #3)) AND #4 |
| #6 | Limited to Chinese and English, from database inception to 31 December 2024, keywords: questionnaire, cross-sectional study, validity, validation study, qualitative study, randomized controlled trial |

***Scopus –* updated search on 13 January 2026**

- Date range from 1 January 2025 to 13 January 2026
- The search strategy and logic (line #1 to #6) remained identical to the original search. The data filter in line #7 was updated to cover the new period from 2025 to 2026.
- 752 new records retrieved

***ProQuest* *(PsycINFO, British Nursing Index)*– original search on 31 December 2024**

| Set | Terms |
| --- | --- |
| #1 | Cyberbullying OR cyberstalking OR cybermob OR deepfake OR doxxing or image-based sexual abuse OR non-image-based sexual abuse OR online grooming OR online initiated physical sexual violence OR online impersonation OR online sexual harassment OR online sexual violence OR online sexual abuse OR online sexual exploitation OR revenge porn OR sexting OR sextortion OR shallowfake OR technology-facilitated sexual violence |
| #2 | Rape OR sexual abuse OR sexual assault OR sexual exploitation OR sexual harassment OR sexual offense OR sexual violence OR intimate partner violence OR spousal violence OR domestic violence OR sexualized material OR commercial sexual exploitation OR child sexual abuse material OR child pornography |
| #3 | Technology OR online OR web OR technology-facilitated OR web-based OR image OR image-based OR video OR WhatsApp OR WeChat OR Tinder OR Blued OR Grindr OR social media OR video game OR livestream OR digital |
| #4 | measure* OR scale* OR tool* OR instrument* OR questionnaire* OR survey* OR validat* OR scale develop* OR index |
| #5 | (#1 OR (#2 AND #3)) AND #4 |
| #6 | Limited to Chinese and English, from database inception to 31 December 2024 |

***ProQuest –* updated search on 13 January 2026**

- Date range from 1 January 2025 to 13 January 2026
- The search strategy and logic (line #1 to #6) remained identical to the original search. The data filter in line #7 was updated to cover the new period from 1 January 2025 to 13 January 2026.
- 1039 new records retrieved

***Web of Science* – original search on 31 December 2024**

| Set | Terms |
| --- | --- |
| #1 | Cyberbullying OR cyberstalking OR cybermob OR deepfake OR doxxing or image-based sexual abuse OR non-image-based sexual abuse OR online grooming OR online initiated physical sexual violence OR online impersonation OR online sexual harassment OR online sexual violence OR online sexual abuse OR online sexual exploitation OR revenge porn OR sexting OR sextortion OR shallowfake OR technology-facilitated sexual violence |
| #2 | ALL (Rape OR sexual abuse OR sexual assault OR sexual exploitation OR sexual harassment OR sexual offense OR sexual violence OR intimate partner violence OR spousal violence OR domestic violence OR sexualized material OR commercial sexual exploitation OR child sexual abuse material OR child pornography) |
| #3 | TS=(“Technology” OR “online” OR “web” OR “technology-facilitated” OR “web-based” OR “image” OR “image-based” OR “video” OR “WhatsApp” OR “WeChat” OR “Tinder” OR “Blued” OR “Grindr” OR “social media” OR “video game” OR “livestream” OR “digital”) |
| #4 | TS=(measure* OR scale* OR tool* OR instrument* OR questionnaire* OR survey* OR validat* OR scale develop* OR index) |
| #5 | (#1 OR (#2 AND #3) AND #4 |
| #6 | Limited to English, from database inception to 31 December 2024, articles and exclude early access articles |

***Web of Science –* updated search on 13 January 2026**

- Date range from 1 January 2025 to 13 January 2026
- The search strategy and logic (line #1 to #6) remained identical to the original search. The data filter in line #7 was updated to cover the new period from 2025 to 2026.
- 1805 new records retrieved

**Chinese National Knowledge Infrastructure- original search on 31 December 2024**

#1

Full Text (數位性暴力) OR Full Text (技術促進的性暴力) OR Full Text (網絡性暴力) OR Full Text (網絡性騷擾) OR Full Text (科技助長性暴力)

#2

Full Text (量表) OR Full Text (量表編製) OR Full Text (信效度評估) OR Full Text (信效度分析) OR Full Text (問卷)

#3

#1 AND #2

**Chinese National Knowledge Infrastructure- updated search on 13 January 2026**

- Date range from 1 January 2025 to 13 January 2026
- The search strategy and logic (line #1 to #3) remained identical to the original search. The data filter in line #3 was updated to cover the new period from 2025 to 2026.
- 0 new record retrieved

**SinoMed- original search on 31 December 2024**

#1

數位性暴力 OR 技術促進的性暴力 OR 網絡性暴力 OR 網絡性騷擾 OR 科技助長性暴力

#2

量表OR量表編製OR信效度評估OR信效度分析OR問卷

#3

#1 AND #2

**SinoMed- updated search on 13 January 2026**

- Date range from 1 January 2025 to 13 January 2026
- The search strategy and logic (line #1 to #3) remained identical to the original search. The data filter in line #3 was updated to cover the new period from 2025 to 2026.
- 0 new record retrieved

**Index to Taiwan Periodical Literature - original search on 31 December 2024**

#1

數位性暴力 OR 技術促進的性暴力 OR 網絡性暴力 OR 網絡性騷擾 OR 科技助長性暴力

#2

量表OR量表編製OR信效度評估OR信效度分析OR問卷

#3

#1 AND #2

**Index to Taiwan Periodical Literature- updated search on 13 January 2026**

- Date range from 1 January 2025 to 13 January 2026
- The search strategy and logic (line #1 to #3) remained identical to the original search. The data filter in line #3 was updated to cover the new period from 2025 to 2026.
- 0 new record retrieved

**Airiti Library- original search on 31 December 2024**

#1

[ALL3]=（影像性暴力）OR [ALL3]= （數位性暴力）OR [ALL3]= （技術促進的性暴力）OR [ALL3]= （技術促進的性暴力）OR [ALL3]= （網絡性暴力）OR [ALL3]= （網絡性騷擾）OR [ALL3]= （科技助長性暴力）

#2

[ALL]= （量表）OR [ALL]= （量表編製）OR [ALL]= （信效度評估）OR [ALL]= （信效度分析）OR [ALL]= （問卷）

#3

#1 AND #2

**Airiti Library - updated search on 13 January 2026**

- Date range from 1 January 2025 to 13 January 2026
- The search strategy and logic (line #1 to #3) remained identical to the original search. The data filter in line #3 was updated to cover the new period from 2025 to 2026.
- 0 new record retrieved
