# Supplementary material for: Measuring Technology-Facilitated Sexual Violence and Abuse: Scoping Review of Existing Measures
Source: J Med Internet Res. 2026 Apr 14;28:e90068. doi: 10.2196/90068 (PMC13125977; doi:10.2196/90068)
Supplement: Multimedia Appendix 4 [file jmir_v28i1e90068_app4.docx]

**Multimedia Appendix 4: All included references**

| **Included studies** | **Geographic region** | **Type of TFSVA** | **Population** | **Outcome measures** |
| --- | --- | --- | --- | --- |
| Baumgartner et al., 2012 [1] | Europe | Online sexual harassment | Adolescents | Behavior |
| Dake et al., 2012 [2] | America | Sexting | Adolescents | Behavior |
| Rice et al., 2012 [3] | America | Sexting | Adolescents | Behavior |
| Temple et al., 2012 [4] | America | Sexting | Adolescents | Behavior |
| Dir et al., 2013 [5] | America | Sexting | Young adults | Behavior |
| Delevi & Weisskirch, 2013 [6] | America | Sexting | Young adults | Behavior |
| Gordon-Messer & Grodzinski, 2013 [7] | America | Sexting | Young adults | Behavior |
| Peskin et al., 2013 [8] | America | Sexting | Adolescents | Behavior |
| Crimmins & Seigfried-Spellar, 2014 [9] | America | Sexting | Young adults | Behavior |
| Drouin & Tobin, 2014 [10] | America | Sexting | Young adults | Behavior and motivation |
| Ritter, 2014 [11] | America | Online sexual harassment | Young adults | Behavior |
| Samimi & Alderson, 2014 [12] | Europe | Sexting | Adults | Behavior |
| Baumgartner et al., 2014 [13] | Europe | Sexting | Adolescents | Behavior |
| Drouin et al., 2014 [14] | America | Sexting | Young adults | Behavior |
| Kerstens & Stol, 2014 [15] | Europe | Image-based sexual abuse | Adolescents | Behavior |
| Yeung et al., 2014 [16] | Australia | Sexting | Young adults | Behavior |
| Ybarra & Mitchell, 2014 [17] | America | Sexting | Adolescents | Behavior |
| Borrajo et al., 2015 [18] | Europe | Cyber sexual dating abuse | Adults | Behavior |
| Champion & Pederse, 2015 [19] | America | Sexting | Young adults | Behavior and attitude |
| Dir & Cyders., 2015 [20] | America | Sexting | Young adults | Behavior |
| Gámez-Guadix et al., 2015 [21] | Europe | Sexting and online sexual harassment | Adults | Behavior |
| Hudson & Fetro, 2015 [22] | America | Sexting | Young adults | Behavior and attitude |
| Hertlein et al., 2015 [23] | America | Sexting | Adults | Behavior |
| McDaniel & Drouin, 2015 [24] | America | Sexting | Adults | Behavior |
| Bianchi et al., 2016 [25] | Europe | Sexting | Adolescents and young adults | Motivation |
| Bobkowski et al., 2016 [26] | America | Online sexual harassment | Adolescents | Behavior |
| Chang et al., 2016 [27] | Asia | Online grooming | Adolescents | Behavior |
| Choi et al., 2016 [28] | America | Sexting | Adolescents | Behavior |
| Currin et al., 2016 [29] | America | Sexting | Adults | Behavior |
| Davis et al., 2016 [30] | America | Sexting | Young adults | Behavior |
| Drouin & Miller, 2016 [31] | America | Online sexual harassment | Adults | Behavior |
| Garcia et al., 2016 [32] | America | Sexting | Adults | Behavior |
| Holt et al., 2016 [33] | America | Sexting | Adolescents | Behavior |
| Lim et al., 2016 [34] | Australia | Sexting | Young adults | Behavior |
| Morelli et al., 2016 [35] | Europe | Sexting | Adolescents and young adults | Behavior |
| Reed et al., 2016 [36] | America | Sexting | Young adults | Behavior |
| Scholes-Balog et al., 2016 [37] | Australia | Sexting | Young adults | Behavior |
| Bianchi et al., 2017 [38] | Europe | Sexting | Adolescents | Motivation |
| Branch et al., 2017 [39] | America | Image-based sexual abuse | Young adults | Attitude |
| Castañeda, 2017 [40] | America | Sexting | Young adults | Behavior |
| García-Gómez, 2017 [41] | Europe | Sexting | Adolescents | Behavior |
| Gámez-Guadix et al., 2017 [42] | Europe | Sexting | Adolescents | Behavior |
| Liong & Cheng, 2017 [43] | Asia | Sexting | Young adults | Attitude |
| Marret & Choo, 2017 [44] | Asia | Online grooming | Adolescents | Behavior |
| Morelli et al., 2017 [45] | America | Sexting | Adolescents and young adults | Behavior |
| Olateunde & Balogun, 2017 [46] | Africa | Sexting | Young adults | Behavior |
| Sánchez et al., 2017 [47] | Europe | Online sexual harassment | Adolescents | Behavior |
| Trub & Starks, 2017 [48] | America | Sexting | Young adults | Behavior |
| Van Oosten & Vandencosch, 2017 [49] | Europe | Sexting | Adolescents | Behavior |
| Van Ouytsel et al., 2017 [50] | Europe | Sexting | Adolescents | Behavior and motivation |
| Villacampa, 2017 [51] | Europe | Sexting | Adolescents | Behavior |
| Buren & Lunde, 2018 [52] | Europe | Sexting | Adolescents | Behavior |
| Cripps & Stermac, 2018 [53] | America | Online sexual harassment | Adults | Behavior |
| Cerón et al., 2018 [54] | America | Sexting | Adolescents | Behavior |
| De Graaf et al., 2018 [55] | Europe | Sexting | Adolescents | Behavior |
| de Santisteban & Gámez-Guadix, 2018 [56] | Europe | Online grooming | Adolescents | Behavior |
| Douglass et al., 2018 [57] | Australia | Online sexual harassment | Young adults | Behavior |
| Galovan et al., 2018 [58] | America | Sexting | Adults | Behavior |
| Gámez-Guadix & Santisteban, 2018 [59] | Europe | Sexting | Adolescents | Behavior |
| Gewirtz-Meydan et al., 2018 [60] | America | Image-based sexual abuse | Adults | Behavior |
| Gámez-Guadix et al., 2018 [61] | Europe | Online grooming | Adolescents | Behavior |
| Gámez-Guadix et al., 2018 [62] | Europe | Online grooming | Adolescents | Behavior |
| Gregg et al., 2018 [63] | America | Sexting | Adolescents | Behavior |
| Kernsmith et al., 2018 [64] | America | Sexting | Adolescents | Behavior |
| Klettke et al., 2018 [65] | Australia | Sexting | Young adults | Behavior |
| Marume et al., 2018 [66] | Africa | Sexting | Adolescents | Behavior |
| Machimbarrena et al., 2018 [67] | Europe | TFSVA | Adolescents | Behavior |
| Marret & Choo, 2018 [68] | Asia | Online sexual harassment | Adolescents | Behavior |
| Medrano et al., 2018 [69] | America | Sexting and online sexual harassment | Young adults | Behavior |
| Patchin & Hinduja, 2018 [70] | America | Sexting | Adolescents | Behavior |
| Rice et al., 2018 [71] | America | Sexting | Adolescents | Behavior |
| Rodríguez-Castro et al., 2018 [72] | Europe | Sexting and cyber sexual dating abuse | Young adults | Behavior and attitude |
| Ševčíková et al., 2018 [73] | Europe | Sexting | Adolescents | Behavior |
| Stanley et al., 2018 [74] | Europe | Sexting | Adolescents | Behavior |
| Watkins et al., 2018 [75] | America | Cyber sexual dating abuse | Adults | Behavior |
| Yoder et al., 2018 [76] | America | Sexting | Adolescents | Behavior and motivation |
| Zetterstro ̈m Dahlqvist & Gillander Ga ̊din, 2018 [77] | Europe | Online grooming | Adolescents | Behavior |
| Atabekova, 2019 [78] | Asia | TFSVA | Adolescents | Behavior |
| Beckmeyer et al., 2019 [79] | America | Sexting | Adolescents | Behavior |
| Brodie et al., 2019 [80] | Europe | Sexting | Adults | Behavior |
| Casas et al., 2019 [81] | Europe | Sexting | Adolescents | Behavior |
| Choi et al., 2019 [82] | America | Sexting | Adolescents | Behavior |
| Currin & Hubach, 2019 [83] | America | Cyber sexual dating abuse | Adults | Motivation |
| Clancy et al., 2019 [84] | Australia | Sexting | Young adults | Behavior |
| Dönmez & Soylu, 2019 [85] | Asia | Online grooming | Adolescents | Behavior |
| Falconer & Humphreys, 2019 [86] | America | Sexting | Adults | Behavior |
| Festl et al., 2019 [87] | Europe | Sexting | Adolescents and young adults | Behavior |
| Florimbio et al., 2019 [88] | America | Sexting | Adults | Behavior |
| Gámez-Guadix et al., 2019 [89] | Europe | Sexting and online grooming | Adolescents | Behavior |
| Gassó et al., 2019 [90] | Europe | Sexting | Young adults | Behavior |
| Ghorashi, 2019 [91] | Asia | Sexting | Adolescents | Behavior |
| Harder et al., 2019 [92] | Europe | Sexting | Young adults | Behavior |
| Ingram et al., 2019 [93] | America | Sexting | Young adults | Attitude |
| Jeanfreau et al., 2019 [94] | America | Sexting | Adults | Behavior and motivation |
| Klein & Cooper, 2019 [95] | America | Online sexual harassment | Young adults | Behavior |
| Klettke et al., 2019 [96] | Australia | Sexting | Young adults | Behavior |
| Maas et al., 2019 [97] | America | Online sexual harassment | Adolescents | Behavior |
| Marengo et al., 2019 [98] | Europe | Sexting | Adolescents | Behavior |
| Ojeda et al., 2019 [99] | Europe | Sexting | Adolescents | Behavior |
| Penado et al., 2019 [100] | Europe | Image-based sexual abuse | Adolescents | Behavior |
| Powell & Henry, 2019 [101] | Australia | TFSVA | Adults | Behavior |
| Powell et al., 2019 [102] | Australia | Image-based sexual abuse | Adults | Attitude |
| Rey et al., 2019 [103] | Europe | Sexting | Adolescents | Behavior |
| Reed et al., 2019 [104] | America | Online sexual harassment | Adolescents | Behavior |
| Ross et al., 2019 [105] | America | Sexting | Young adults | Behavior |
| Tang et al., 2019 [106] | Europe | Online sexual harassment | Adolescents and adults | Behavior |
| Titchen et al., 2019 [107] | America | Sexting | Adolescents | Behavior |
| Yépez-Tito et al., 2019 [108] | Europe | Sexting | Adolescents | Behavior |
| Baiden et al., 2020 [109] | Africa | Sexting | Adolescents | Behavior |
| Calvete et al., 2020 [110] | Europe | Cyber sexual dating abuse and online grooming | Adolescents | Behavior |
| Clancy et al., 2020 [111] | Australia | Sexting | Young adults | Behavior |
| Currin et al., 2020 [112] | America | Sexting | Adults | Behavior |
| Corcoran et al., 2020 [113] | America | Online sexual harassment | Young adults | Behavior |
| Confalonieri et al., 2020 [114] | Europe | Sexting | Adolescents | Behavior and attitude |
| Cornelius et al., 2020 [115] | America | Sexting | Young adults | Behavior |
| Cavalcanti et al., 2020 [116] | America | Cyber sexual dating abuse | Adults | Behavior |
| De Abreu Silva et al., 2020 [117] | America | Sexting | Young adults | Behavior |
| Dolev-cohen & Ricon, 2020 [118] | Asia | Sexting | Adolescents | Behavior |
| Dodaj et al., 2020 [119] | Europe | Sexting | Adolescents | Behavior |
| Florimbio et al., 2020 [120] | America | Sexting | Adults | Behavior |
| Gassó et al., 2020 [121] | Europe | Sexting | Young adults | Behavior |
| Geeng et al., 2020 [122] | America | Sexting | Adults | Behavior |
| Gil-Llario et al., 2020a [123] | Europe | Sexting | Adolescents | Behavior, attitude, and motivation |
| Gil-Llario et al., 2020b [124] | Europe | Sexting | Adolescents | Behavior, attitude, and motivation |
| Gómez-García et al., 2020 [125] | Europe | Sexting | Young adults | Behavior |
| Kim et al., 2020 [126] | America | Sexting | Adolescents | Behavior |
| Lucić et al., 2020 [127] | Europe | TFSVA | Adolescents | Behavior |
| Molla-Esparza et al., 2020 [128] | Europe | Sexting | Adolescents | Behavior |
| Morelli et al., 2020 [129] | Multi-countries | Sexting | Adolescents and young adults | Behavior |
| Oswald et al., 2020 [130] | America | Image-based sexual abuse | Adults | Motivation |
| Paquette & Cortoni, 2020 [131] | America | Online sexual harassment | Adolescents | Behavior |
| Reed et al., 2020 [132] | America | Sexting | Adolescents | Behavior and motivation |
| Snaychuk & O’Neill, 2020 [133] | America | TFSVA | Young adults | Behavior |
| Mckinlay & Lavis, 2020 [134] | Australia | Image-based sexual abuse | Adults | Behavior |
| Muncaster & Ohlsson, 2020 [135] | America | Sexting | Adults | Behavior |
| Niehuis et al., 2020 [136] | America | Image-based sexual abuse | Young adults | Behavior |
| Turban et al., 2020 [137] | America | Sexting | Adults | Behavior |
| Yépez-Tito et al., 2020 [138] | Europe | Sexting | Adolescents | Behavior |
| Yu & Zheng, 2020 [139] | Asia | Sexting | Young adults | Behavior |
| Buric et al., 2021 [140] | Europe | Sexting | Adolescents | Behavior |
| Brown & Hegarty, 2021 [141] | Australia | Cyber sexual dating abuse | Adolescents | Behavior |
| del Rey et al., 2021 [142] | Europe | Sexting | Adolescents | Behavior |
| Gámez-Guadix et al., 2021a [143] | Europe | Online grooming | Adolescents | Behavior |
| Gámez-Guadix et al. 2021b [144] | Europe | Online sexual harassment | Adolescents | Behavior |
| Gil-Llario et al., 2021 [145] | Europe and America | Sexting | Adolescents | Behavior, attitude, and motivation |
| García-González et al., 2021 [146] | Europe and America | Sexting | Young adults | Behavior |
| Hinduja & Patchin, 2021 [147] | America | Cyber sexual dating abuse | Adolescents | Behavior |
| Lopex-Cepero et al., 2021 [148] | America | Cyber sexual dating abuse | Adolescents | Behavior |
| Barroso et al., 2021 [149] | Europe | Sexting | Adolescents | Behavior |
| Boer et al., 2021 [150] | Europe | Sexting | Adolescents | Behavior |
| Beckmeyer et al., 2021 [151] | America | Sexting | Young adults | Behavior |
| Bianchi at el., 2021a [152] | Europe | Sexting | Adolescents | Motivation |
| Bianchi et al., 2021b [153] | Europe | Sexting | Adolescents and young adults | Behavior |
| Bianchi et al., 2021c [154] | Europe | Sexting | Adolescents and young adults | Motivation |
| Brown et al., 2021 [155] | Australia | Cyber sexual dating abuse | Young adults | Behavior |
| Calvete et al., 2021 [156] | Europe | Sexting and online grooming | Adults | Behavior |
| Champion et al., 2021 [157] | America | TFSVA | Adolescents and adults | Behavior |
| Clancy et al., 2021a [158] | Australia | Sexting | Young adults | Behavior |
| Clancy et al., 2021b [159] | Australia and America | Sexting | Young adults | Behavior |
| Copp et al., 2021 [160] | America | Online sexual harassment | Adolescents | Behavior |
| Courtice et al., 2021 [161] | America | TFSVA | Adults | Behavior |
| Durmus & Solak, 2021 [162] | Asia | Sexting | Adults | Behavior |
| Foody et al., 2021 [163] | Europe | Sexting | Adolescents | Behavior |
| Gassó & Gómez-Durán, 2021 [164] | Europe | Sexting | Young adults | Behavior |
| Gassó et al., 2021a [165] | Europe | Sexting | Young adults | Behavior |
| Gassó et al., 2021b [166] | Europe | Sexting | Young adults | Behavior |
| Hernández et al., 2021 [167] | Europe | Sexting and online grooming | Adolescents | Behavior |
| Howard et al., 2021 [168] | Australia | Sexting | Young adults | Behavior |
| Hunter et al., 2021 [169] | Australia | Sexting | Adolescents | Behavior |
| Kafaee & Kohut, 2021 [170] | America | Sexting | Adults | Behavior |
| Longobardi et al., 2021 [171] | Europe | Online sexual harassment | Adolescents | Behavior |
| Lu et al., 2021 [172] | America | Sexting | Adolescents | Behavior |
| Maas et al., 2021 [173] | America | Image-based sexual abuse | Young adults | Behavior |
| Matotek et al., 2021 [174] | Australia | Sexting | Adults | Behavior |
| Mori et al., 2021 [175] | America | Sexting | Adolescents | Behavior |
| Morelli et al., 2021 [176] | Multi-countries | Sexting | Adolescents and young adults | Behavior |
| Reer et al., 2021 [177] | Europe | Online sexual harassment | Adolescents and adults | Behavior and motivation |
| Resett et al., 2021 [178] | America | Sexting and online grooming | Adolescents | Behavior |
| Rodríguez-Castro et al., 2021 [179] | Europe | Sexting and cyber sexual dating abuse | Adolescents | Behavior |
| Ståhl & Dennhag, 2021 [180] | Europe | Online sexual harassment | Adolescents | Behavior |
| Van Ouytsel et al., 2021a [181] | America | Sexting | Adolescents | Behavior |
| Van Ouytsel et al., 2021b [182] | Europe | Sexting | Adults | Behavior |
| Yépez-Tito et al., 2021 [183] | Europe | Sexting | Adolescents | Behavior |
| Walker et al., 2021 [184] | Europe | Image-based sexual abuse | Young adults | Behavior |
| Wachs et al., 2021 [185] | America | Sexting | Adolescents | Behavior |
| Widman et al., 2021 [186] | America | Sexting | Adolescents | Behavior |
| Buchanan & Mahoney, 2022 [187] | Europe | Online sexual harassment | Adolescents | Behavior |
| Belotti et al., 2022 [188] | Europe | Online sexual harassment | Adolescents | Behavior |
| Champion et al., 2022 [189] | America | TFSVA | Adults | Behavior |
| Calvete et al., 2022 [190] | Europe | Online grooming | Adolescents | Behavior |
| Califano et al., 2022 [191] | Europe | Sexting | Adults | Behavior and motivation |
| Caponnetto et al., 2022 [192] | Europe | Sexting | Young adults and adults | Behavior and motivation |
| Currin, 2022 [193] | America | Sexting | Adults | Motivation |
| Currin et al., 2022 [194] | America | Sexting | Adults | Motivation |
| Díaz-Aguado & Martínez-Arias, 2022 [195] | Europe | Cyber sexual dating abuse and online sexual harassment | Adolescents | Behavior |
| Dodaj et al., 2022 [196] | Europe | Sexting | Young adults | Behavior |
| Dolev-Cohen & Ricon, 2022a [197] | Asia | Sexting | Adults | Behavior |
| Dolev-Cohen & Ricon, 2022b [198] | Asia | Sexting | Adults | Behavior |
| Dolev-Cohen & Ricon, 2022c [199] | Asia | Sexting | Adults | Behavior |
| Fissel et al., 2022 [200] | America | Cyber sexual dating abuse | Adults | Behavior |
| Jeanfreau et al., 2022 [201] | America | Sexting | Adults | Behavior and motivation |
| Kokkinos & Krommida, 2022 [202] | Europe | Sexting | Adults | Behavior |
| Gámez-Guadix et al., 2022 [203] | Europe | Image-based sexual abuse | Adolescents | Behavior |
| Pasca et al., 2022 [204] | Europe | Online grooming | Adolescents | Behavior |
| Qu et al., 2022 [205] | America and China | Sexting | Young adults | Behavior |
| Resett et al., 2022 [206] | America | Sexting and online grooming | Adolescents | Behavior |
| Romero-Rodríguez et al., 2022 [207] | Europe | Sexting | Young adults | Behvaior |
| Sparks, 2022 [208] | Europe | Image-based sexual abuse | Adults | Behavior |
| Zagloul et al., 2022 [209] | Africa | TFSVA | Adults | Behavior |
| Barrense-Dias et al., 2022 [210] | Europe | Sexting | Adolescents | Behavior |
| Bielčiková & Hollá et al., 2022 [211] | Europe | Sexting | Adolescents | Behavior |
| Bragard et al., 2022 [212] | America | Sexting | Adolescents | Behavior |
| Buren et al., 2022 [213] | Europe | Sexting | Adolescents | Behavior |
| Corcoran et al., 2022 [214] | America | Sexting | Adults | Behavior |
| Crapolicchio et al., 2022 [215] | Europe | Sexting | Adults | Behavior |
| Dardis & Richards, 2022 [216] | America | Image-based sexual abuse | Young adults | Behavior |
| Finkelhor et al., 2022 [217] | America | Online sexual harassment | Young adults | Behavior |
| Garner et al., 2022 [218] | America | Sexting | Adults | Behavior |
| Greer et al., 2022 [219] | America | Sexting | Young adults | Behavior |
| Gargano et al., 2022 [220] | Europe | Sexting | Adolescents | Behavior and motivation |
| Guest et al., 2022 [221] | America | Sexting | Young adults | Behavior |
| Huiskes et al., 2022 [222] | Europe | TFSVA | Adults | Attitude |
| Kamar et al., 2022 [223] | Asia | Online grooming | Adults | Behavior |
| Maes & Vandenbosch, 2022 [224] | Europe | Sexting | Adolescents | Behavior |
| Marcum et al., 2022 [225] | America | Image-based sexual abuse | Young adults | Behavior |
| Ng et al., 2022 [226] | America | Sexting | Adolescents | Behavior |
| Noorishad & Trottier, 2022 [227] | America | Sexting | Adolescents and adults | Behavior |
| Powell et al., 2022a [228] | Europe and Australia | Image-based sexual abuse | Adolescents and adults | Behavior |
| Powell et al., 2022b [229] | Europe and Australia | Image-based sexual abuse | Adolescents and adults | Behavior |
| Paquette et al., 2022 [230] | America | Sexting | Adolescents | Behavior |
| Rollero & Pagliaro, 2022 [231] | Europe | Image-based sexual abuse | Adults | Behavior |
| Durán & Rodríguez-Domínguez, 2023 [232] | Europe | Online sexual harassment | Adults | Behavior |
| Harper et al., 2023 [233] | Europe | Image-based sexual abuse | Adults | Attitude |
| Hussain et al., 2023 [234] | Asia | Sexting | Adults | Motivation |
| Bianchi et al., 2023 [235] | Europe | Sexting | Young adults | Behavior |
| Beckmeyer, 2023 [236] | America | Sexting | Young adults | Behavior |
| Bogner et al., 2023 [237] | Europe | Sexting | Adolescents | Behavior |
| Bonfanti et al., 2023 [238] | Europe | Sexting | Adults | Motivation |
| Borgogna et al., 2023 [239] | America | Sexting | Young adults | Behavior |
| Brighi et al., 2023 [240] | Europe | Sexting | Young adults and adults | Behavior |
| Cross et al., 2023 [241] | Australia | Sexting | Adults | Behavior |
| Currin et al., 2023 [242] | America | Sexting | Adults | Motivation |
| Chan & Wu-Ouyang, 2023 [243] | Asia | Sexting | Adults | Behavior and motivation |
| Checkalski et al., 2023 [244] | America | Sexting | Young adults | Motivation |
| Choi et al., 2023 [245] | Asia | Online sexual harassment | Adolescents | Behavior |
| Cuccì et al., 2023 [246] | Europe | Sexting | Adolescents | Behavior |
| Dodaj & Sesar, 2023 [247] | Europe | Sexting | Adults | Behavior and attitude |
| Dodaj & Sesar, 2023 [248] | Europe | Sexting | Young adults | Behavior |
| Dodaj et al., 2023 [249] | Europe | Sexting | Young adults | Behavior |
| Dolev-Cohen, 2023 [250] | Asia | Sexting | Adolescents | Behavior and motivation |
| Eaton et al., 2023 [251] | America | Sexting | Adults | Behavior |
| Finkelhor et al., 2023 [252] | America | Online sexual harassment | Young adults | Behavior |
| Falconer et al., 2023 [253] | America | Sexting | Adults | Behavior |
| Flynn et al., 2023 [254] | Europe | Image-based sexual abuse | Adults | Behavior |
| Foody et al., 2023 [255] | Europe | Sexting | Adolescents | Behavior |
| Garrido-Macías et al., 2023 [256] | Europe | Sexting | Adults | Behavior |
| Goh et al., 2023 [257] | Asia | Sexting | Young adults | Behavior and motivation |
| Gesselman et al., 2023 [258] | America | TFSVA | Adults | Behavior |
| Hassan et al., 2023 [259] | Asia | Sexting | Young adults | Attitude |
| Holfeld et al., 2023 [260] | America | Sexting | Adolescents | Behavior |
| Hong et al., 2023 [261] | Asia | Online sexual harassment | Adolescents | Behavior |
| Huntington & Rhoades, 2023 [262] | America | Sexting | Adolescents | Behavior |
| Huang et al., 2023 [263] | Asia | Online sexual harassment | Adolescents | Behavior |
| Kamar et al., 2023 [264] | Asia | Online grooming | Adults | Behavior and motivation |
| Karasavva et al., 2023 [265] | America | Image-based sexual abuse | Young adults | Attitude |
| Kokkinos et al., 2023 [266] | Europe | Sexting | Young adults | Behavior |
| Lunde et al., 2023 [267] | Europe | Sexting | Adolescents | Behavior |
| Maes et al., 2023 [268] | Europe | Sexting | Adolescents and young adults | Behavior |
| Molla-Esparza et al., 2023a [269] | Europe | Sexting | Adolescents | Behavior |
| Molla-Esparza et al., 2023b [270] | Europe | Sexting | Adolescents | Behavior |
| Morelli et al., 2023a [271] | America | Sexting | Adolescents | Behavior |
| Morelli et al., 2023b [272] | Europe | Sexting | Adults | Behavior |
| Mussap et al., 2023a [273] | Australia | Sexting | Adults | Behavior |
| Mussap et al., 2023b [274] | Australia | Sexting | Adults | Behavior |
| Okumu et al., 2023 [275] | Africa | Sexting | Adolescents | Behavior |
| Parti et al., 2023 [276] | America | Sexting | Young adults | Behavior |
| Pedersen et al., 2023 [277] | Europe | Image-based sexual abuse | Adolescents | Behavior |
| Pistoni et al., 2023 [278] | Europe | Sexting | Adolescents | Behavior |
| Ragona et al., 2023 [279] | Europe | Sexting | Adolescents and young adults | Behavior and motivation |
| Rollero et al., 2023 [280] | Europe | Sexting and image-based sexual abuse | Adults | Behavior |
| Sesar et al., 2023 [281] | Europe | Sexting | Adults | Behavior |
| Karasavva et al., 2023 [282] | America | Online sexual harassment | Young adults | Behavior and motivation |
| Ricon & Dolev-Cohen, 2023 [283] | Asia | Sexting | Adults | Behavior |
| Schokkenbroek et al., 2023 [284] | Europe | Sexting | Adults | Behavior |
| Sparks et al., 2023 [285] | Europe | Image-based sexual abuse | Young adults | Behavior |
| Sciacca et al., 2023 [286] | Europe | Image-based sexual abuse | Adolescents | Behavior |
| Almeida & Barreiros, 2024 [287] | Europe | Online grooming | Adolescents | Behavior |
| Cary et al., 2024 [288] | America | Online sexual harassment | Young adults | Behavior |
| Damra et al., 2024 [289] | Asia | Cyber sexual dating abuse | Adults | Behavior |
| Kowalski & Thompson, 2024 [290] | America | Image-based sexual abuse | Not specified | Behavior |
| Maes et al., 2024 [291] | Europe | Sexting | Young adults | Behavior |
| Martínez-Bacaicoa et al., 2024 [292] | Europe | TFSVA | Adults | Behavior |
| Martínez Soto et al., 2024 [293] | Europe | Cyber sexual dating abuse | Adolescents | Behavior |
| Muñoz-Fernández et al., 2024 [294] | Europe | Online sexual harassment | Adolescents | Behavior |
| Orsolini et al., 2024 [295] | Europe | TFSVA | Young adults | Behvaior |
| Pak et al., 2024 [296] | Asia | TFSVA | Adults | Behavior and attitude |
| Antoniado & Kokkinos., 2025 [297] | Europe | Online sexual harassment | Adults | Behavior |
| Colburn et al., 2025 [298] | America | Image-based sexual abuse | Young adults | Behavior |
| Dardis et al., 2025 [299] | America | Image-based sexual abuse | Young adults | Behavior and attitude |
| Finkelhor et al., 2025 [300] | America | Image-based sexual abuse | Young adults | Behavior |
| Gewirtz-Meydan et al., 2025 [301] | America | Image-based sexual abuse | Young adults | Behavior |
| Kokkinos et al., 2025 [302] | Europe | Image-based sexual abuse | Young adults | Behavior, attitude, and motivation |
| Kokkinos et al., 2025 [303] | Europe | TFSVA | Adults | Behavior |
| Pijlman et al., 2025 [304] | Europe | Image-based sexual abuse | Adolescents and young adults | Behavior |
| Turner et al., 2025 [305] | America | Image-based sexual abuse | Young adults | Behavior |
| Umbach & Henry, 2025 [306] | Australia, America, Europe, and Asia | Image-based sexual abuse | Not specified | Behavior, attitude, and motivation |
| Maher et al., 2025 [307] | America | Image-based sexual abuse | Young adults | Behavior |
| Wongsomboon et al., 2025 [308] | America | Cyber sexual dating abuse | Adolescents | Behavior |
| Mathews et al., 2025 [309] | Australia | Online grooming | Adolescents and young adults | Behavior |
| Walsh et al., 2025a [310] | Australia | Online grooming | Adolescents and young adults | Behavior |
| Walsh et al., 2025b [311] | America | Online grooming | Young adults | Behavior |
| Mathews et al., 2026 [312] | America | Online grooming | Adolescents and young adults | Behavior |
| Bhuptani et al., 2025 [313] | America | Online sexual harassment | Adults | Behavior |
| Jannite et al., 2025 [314] | Asia | Online sexual harassment | Young adults | Behavior |
| Ahn et al., 2025 [315] | America | Sexting | Adults | Behavior |
| Palomino-Ccasa et al., 2025 [316] | America | Sexting | Adults | Behavior |
| Ozdag et al., 2025 [317] | Europe | TFSVA | Adolescents | Behavior |
| Ragona et al., 2025 [318] | Europe | Cyber sexual dating abuse | Young adults | Behavior |
| Resett et al., 2025 [319] | America | Sexting and online grooming | Adolescents | Behavior |

1. Baumgartner, S.E., et al., *Identifying teens at risk: Developmental pathways of online and offline sexual risk behavior.* Pediatrics, 2012. **130**(6): p. e1489-e1496.

2. Dake, J.A., et al., *Prevalence and Correlates of Sexting Behavior in Adolescents.* American journal of sexuality education, 2012. **7**(1): p. 1-15.

3. Rice, E., et al., *Sexually Explicit Cell Phone Messaging Associated With Sexual Risk Among Adolescents.* Pediatrics (Evanston), 2012. **130**(4): p. 667-673.

4. Temple, J.R., et al., *Teen sexting and its association with sexual behaviors.* Archives of Pediatrics and Adolescent Medicine, 2012. **166**(9): p. 828-833.

5. Dir, A.L., M.A. Cyders, and A. Coskunpinar, *From the bar to the bed via mobile phone: A first test of the role of problematic alcohol use, sexting, and impulsivity-related traits in sexual hookups.* Computers in Human Behavior, 2013. **29**(4): p. 1664-1670.

6. Delevi, R. and R.S. Weisskirch, *Personality factors as predictors of sexting.* Computers in Human Behavior, 2013. **29**(6): p. 2589-2594.

7. Gordon-Messer, D., et al., *Sexting among young adults.* Journal of Adolescent Health, 2013. **52**(3): p. 301-306.

8. Peskin, M.F., et al., *Prevalence and patterns of sexting among ethnic minority urban high school students.* Cyberpsychology, Behavior, and Social Networking, 2013. **16**(6): p. 454-459.

9. Crimmins, D.M. and K.C. Seigfried-Spellar, *Peer attachment, sexual experiences, and risky online behaviors as predictors of sexting behaviors among undergraduate students.* Computers in Human Behavior, 2014. **32**: p. 268-275.

10. Drouin, M. and E. Tobin, *Unwanted but consensual sexting among young adults: Relations with attachment and sexual motivations.* Computers in Human Behavior, 2014. **31**(1): p. 412-418.

11. Ritter, B.A., *Deviant Behavior in Computer-Mediated Communication: Development and Validation of a Measure of Cybersexual Harassment.* Journal of computer-mediated communication, 2014. **19**(2): p. 197-214.

12. Samimi, P. and K.G. Alderson, *Sexting among undergraduate students.* Computers in Human Behavior, 2014. **31**(1): p. 230-241.

13. Baumgartner, S.E., et al., *Does country context matter? Investigating the predictors of teen sexting across Europe.* Computers in Human Behavior, 2014. **34**: p. 157-164.

14. Drouin, M., E. Tobin, and K. Wygant, *“Love the Way You Lie”: Sexting deception in romantic relationships.* Computers in human behavior, 2014. **35**: p. 542-547.

15. Kerstens, J. and W. Stol, *Receiving online sexual requests and producing online sexual images: The multifaceted and dialogic nature of adolescents' online sexual interactions.* Cyberpsychology, 2014. **8**(1).

16. Yeung, T.H., et al., *Prevalence, correlates and attitudes towards sexting among young people in Melbourne, Australia.* Sexual health, 2014. **11**(4): p. 332-339.

17. Ybarra, M.L. and K.J. Mitchell, *"sexting" and its relation to sexual activity and sexual risk behavior in a national survey of adolescents.* Journal of Adolescent Health, 2014. **55**(6): p. 757-764.

18. Borrajo, E., M. Gámez-Guadix, and E. Calvete, *Cyber Dating Abuse: Prevalence, Context, and Relationship with Offline Dating Aggression.* Psychological reports, 2015. **116**(2): p. 565-585.

19. Champion, A.R. and C.L. Pedersen, *Investigating differences between sexters and non-sexters on attitudes, subjective norms, and risky sexual behaviours.* Canadian Journal of Human Sexuality, 2015. **24**(3): p. 205-214.

20. Dir, A.L. and M.A. Cyders, *Risks, Risk Factors, and Outcomes Associated with Phone and Internet Sexting Among University Students in the United States.* Archives of Sexual Behavior, 2015. **44**(6): p. 1675-1684.

21. Gámez-Guadix, M., et al., *Prevalence and Association of Sexting and Online Sexual Victimization Among Spanish Adults.* Sexuality Research and Social Policy, 2015. **12**(2): p. 145-154.

22. Hudson, H.K. and J.V. Fetro, *Sextual activity: Predictors of sexting behaviors and intentions to sext among selected undergraduate students.* Computers in human behavior, 2015. **49**: p. 615-622.

23. Hertlein, K.M., C. Shadid, and S.M. Steelman, *Exploring Perceptions of Acceptability of Sexting in Same-Sex, Bisexual, Heterosexual Relationships and Communities.* Journal of Couple and Relationship Therapy, 2015. **14**(4): p. 342-357.

24. McDaniel, B.T. and M. Drouin, *Sexting among married couples: Who is doing it, and are they more satisfied?* Cyberpsychology, Behavior, and Social Networking, 2015. **18**(11): p. 628-634.

25. Bianchi, D., et al., *Psychometric properties of the Sexting Motivations Questionnaire for adolescents and young adults.* Rassegna di Psicologia, 2016. **33**(3): p. 5-18.

26. Bobkowski, P.S., A. Shafer, and R.R. Ortiz, *Sexual intensity of adolescents' online self-presentations: Joint contribution of identity, media consumption, and extraversion.* Computers in Human Behavior, 2016. **58**: p. 64-74.

27. Chang, F.C., et al., *Predictors of unwanted exposure to online pornography and online sexual solicitation of youth.* J Health Psychol, 2016. **21**(6): p. 1107-18.

28. Choi, H., J. Van Ouytsel, and J.R. Temple, *Association between sexting and sexual coercion among female adolescents.* Journal of Adolescence, 2016. **53**: p. 164-168.

29. Currin, J.M., et al., *Explicitly Pressing Send: Impact of Sexting on Relationship Satisfaction.* American Journal of Family Therapy, 2016. **44**(3): p. 143-154.

30. Davis, M.J., et al., *I want your sext: Sexting and sexual risk in emerging adult minority men.* AIDS Education and Prevention, 2016. **28**(2): p. 138-152.

31. Drouin, M. and D.A. Miller, *Online erotica usage as a mediator between internet addiction and engagement in risky online sexual behaviors.* Cyberpsychology, 2016. **10**(3).

32. Garcia, J.R., et al., *Sexting among singles in the USA: Prevalence of sending, receiving, and sharing sexual messages and images.* Sexual Health, 2016. **13**(5): p. 428-435.

33. Holt, T.J., et al., *Identifying Predictors of Unwanted Online Sexual Conversations Among Youth Using a Low Self-Control and Routine Activity Framework.* Journal of Contemporary Criminal Justice, 2016. **32**(2): p. 108-128.

34. Lim, M.S.C., et al., *Exploring attitudes towards sexting of young people: A cross-sectional study.* Sexual Health, 2016. **13**(6): p. 530-535.

35. Morelli, M., et al., *Sexting, psychological distress and dating violence among adolescents and young adults.* Psicothema, 2016. **28**(2): p. 137-142.

36. Reed, L.A., R.M. Tolman, and L.M. Ward, *Snooping and Sexting: Digital Media as a Context for Dating Aggression and Abuse Among College Students.* Violence Against Women, 2016. **22**(13): p. 1556-1576.

37. Scholes-Balog, K., N. Francke, and S. Hemphill, *Relationships Between Sexting, Self-Esteem, and Sensation Seeking Among Australian Young Adults.* Sexualization, media and society, 2016. **2**(2).

38. Bianchi, D., et al., *Sexting as the mirror on the wall: Body-esteem attribution, media models, and objectified-body consciousness.* Journal of Adolescence, 2017. **61**: p. 164-172.

39. Branch, K., et al., *Revenge Porn Victimization of College Students in the United States: An Exploratory Analysis.* International journal of cyber criminology, 2017. **11**(1): p. 128-142.

40. Castañeda, D.M., *Sexting and Sexuality in Romantic Relationships Among Latina/o Emerging Adults.* American Journal of Sexuality Education, 2017. **12**(2): p. 120-135.

41. García-Gómez, A., *Teen girls and sexual agency: exploring the intrapersonal and intergroup dimensions of sexting.* Media, Culture and Society, 2017. **39**(3): p. 391-407.

42. Gámez-Guadix, M., P. de Santisteban, and S. Resett, *Sexting among Spanish adolescents: Prevalence and personality profiles.* Psicothema, 2017. **29**(1): p. 29-34.

43. Liong, M. and G.H.L. Cheng, *Sext and gender: examining gender effects on sexting based on the theory of planned behaviour.* Behaviour and Information Technology, 2017. **36**(7): p. 726-736.

44. Marret, M.J. and W.Y. Choo, *Factors associated with online victimisation among Malaysian adolescents who use social networking sites: a cross-sectional study.* BMJ Open, 2017. **7**(6): p. e014959.

45. Morelli, M., et al., *Sexting Behaviors and Cyber Pornography Addiction Among Adolescents: the Moderating Role of Alcohol Consumption.* Sexuality Research and Social Policy, 2017. **14**(2): p. 113-121.

46. Olatunde, O. and F. Balogun, *Sexting: Prevalence, predictors, and associated sexual risk behaviors among postsecondary school young people in Ibadan, Nigeria.* Frontiers in Public Health, 2017. **5**(MAY).

47. Sánchez, V., N. Muñoz-Fernández, and E. Vega-Gea, *Peer sexual cybervictimization in adolescents: Development and validation of a scale.* International journal of clinical and health psychology, 2017. **17**(2): p. 171-179.

48. Trub, L. and T.J. Starks, *Insecure attachments: Attachment, emotional regulation, sexting and condomless sex among women in relationships.* Computers in Human Behavior, 2017. **71**: p. 140-147.

49. van Oosten, J.M.F. and L. Vandenbosch, *Sexy online self-presentation on social network sites and the willingness to engage in sexting: A comparison of gender and age.* Journal of Adolescence, 2017. **54**: p. 42-50.

50. Van Ouytsel, J., et al., *Sexting: adolescents’ perceptions of the applications used for, motives for, and consequences of sexting.* Journal of Youth Studies, 2017. **20**(4): p. 446-470.

51. Villacampa, C., *Teen sexting: Prevalence, characteristics and legal treatment.* International Journal of Law, Crime and Justice, 2017. **49**: p. 10-21.

52. Burén, J. and C. Lunde, *Sexting among adolescents: A nuanced and gendered online challenge for young people.* Computers in Human Behavior, 2018. **85**: p. 210-217.

53. Cripps, J. and L. Stermac, *Cyber-sexual violence and negative emotional states among women in a Canadian University.* International Journal of Cyber Criminology, 2018. **12**(1): p. 171-186.

54. Cerón, M.A., L.B. Eisman, and F.F. Palomares, *Grooming, cyberbullying and sexting in Chile according of sex and school management or administrative dependency.* Revista Chilena de Pediatria, 2018. **89**(3): p. 352-360.

55. De Graaf, H., et al., *Offline and Online Sexual Risk Behavior among Youth in the Netherlands: Findings from “Sex under the Age of 25”.* Frontiers in Public Health, 2018. **6**.

56. de Santisteban, P. and M. Gámez-Guadix, *Prevalence and Risk Factors Among Minors for Online Sexual Solicitations and Interactions With Adults.* Journal of Sex Research, 2018. **55**(7): p. 939-950.

57. Douglass, C.H., et al., *Correlates of in-person and technology-facilitated sexual harassment from an online survey among young Australians.* Sex Health, 2018. **15**(4): p. 361-365.

58. Galovan, A.M., M. Drouin, and B.T. McDaniel, *Sexting profiles in the United States and Canada: Implications for individual and relationship well-being.* Computers in Human Behavior, 2018. **79**: p. 19-29.

59. Gámez-Guadix, M. and P. de Santisteban, *“Sex Pics?”: Longitudinal Predictors of Sexting Among Adolescents.* Journal of Adolescent Health, 2018. **63**(5): p. 608-614.

60. Gewirtz-Meydan, A., et al., *The complex experience of child pornography survivors.* Child Abuse Negl, 2018. **80**: p. 238-248.

61. Gámez-Guadix, M., et al., *Persuasion strategies and sexual solicitations and interactions in online sexual grooming of adolescents: Modeling direct and indirect pathways.* J Adolesc, 2018. **63**: p. 11-18.

62. Gámez-Guadix, M., P. De Santisteban, and M. Alcazar, *The Construction and Psychometric Properties of the Questionnaire for Online Sexual Solicitation and Interaction of Minors With Adults.* Sex Abuse, 2018. **30**(8): p. 975-991.

63. Gregg, D., et al., *Sexting Rates and Predictors From an Urban Midwest High School.* Journal of School Health, 2018. **88**(6): p. 423-433.

64. Kernsmith, P.D., B.G. Victor, and J.P. Smith-Darden, *Online, Offline, and Over the Line: Coercive Sexting Among Adolescent Dating Partners.* Youth and Society, 2018. **50**(7): p. 891-904.

65. Klettke, B., et al., *Sexting and mental health: A study of indian and australian young adults.* Cyberpsychology, 2018. **12**(2).

66. Marume, A., J. Maradzika, and J. January, *Adolescent Sexting and Risky Sexual Behaviours in Zimbabwe: A Cross-Sectional Study.* Sexuality and Culture, 2018. **22**(3): p. 931-941.

67. Machimbarrena, J.M., et al., *Internet risks: An overview of victimization in cyberbullying, cyber dating abuse, sexting, online grooming and problematic internet use.* International Journal of Environmental Research and Public Health, 2018. **15**(11).

68. Marret, M.J. and W.Y. Choo, *Victimization After Meeting With Online Acquaintances: A Cross-Sectional Survey of Adolescents in Malaysia.* J Interpers Violence, 2018. **33**(15): p. 2352-2378.

69. Medrano, J.L.J., F. Lopez Rosales, and M. Gámez-Guadix, *Assessing the Links of Sexting, Cybervictimization, Depression, and Suicidal Ideation Among University Students.* Archives of Suicide Research, 2018. **22**(1): p. 153-164.

70. Patchin, J.W. and S. Hinduja, *Sextortion Among Adolescents: Results From a National Survey of U.S. Youth.* Sex Abuse, 2020. **32**(1): p. 30-54.

71. Rice, E., et al., *Associations Between Sexting Behaviors and Sexual Behaviors Among Mobile Phone-Owning Teens in Los Angeles.* Child Development, 2018. **89**(1): p. 110-117.

72. Rodríguez-Castro, Y., et al., *From sexting to cybercontrol among dating teens in Spain: An analysis of their arguments.* Revista Latinoamericana de Psicologia, 2018. **50**(3): p. 170-178.

73. Ševčíková, A., L. Blinka, and K. Daneback, *Sexting as a predictor of sexual behavior in a sample of Czech adolescents.* European Journal of Developmental Psychology, 2018. **15**(4): p. 426-437.

74. Stanley, N., et al., *Pornography, Sexual Coercion and Abuse and Sexting in Young People’s Intimate Relationships: A European Study.* Journal of interpersonal violence, 2018. **33**(19): p. 2919-2944.

75. Watkins, L.E., R.C. Maldonado, and D. DiLillo, *The Cyber Aggression in Relationships Scale: A New Multidimensional Measure of Technology-Based Intimate Partner Aggression.* Assessment (Odessa, Fla.), 2018. **25**(5): p. 608-626.

76. Yoder, J., J. Hansen, and M. Precht, *Correlates and outcomes associated with sexting among justice involved youth: The role of developmental adversity, emotional disinhibitions, relationship context, and dating violence.* Children and Youth Services Review, 2018. **94**: p. 493-499.

77. Zetterström Dahlqvist, H. and K. Gillander Gådin, *Online sexual victimization in youth: predictors and cross-sectional associations with depressive symptoms.* Eur J Public Health, 2018. **28**(6): p. 1018-1023.

78. Atabekova, A., *Technology-facilitated harm to individuals and society: Cases of minor’s self-produced sexual content in Russia.* Journal of Critical Reviews, 2019. **6**(6): p. 410-415.

79. Beckmeyer, J.J., et al., *Characteristics of Adolescent Sexting: Results from the 2015 National Survey of Sexual Health and Behavior.* Journal of Sex and Marital Therapy, 2019. **45**(8): p. 767-780.

80. Brodie, Z.P., C. Wilson, and G.G. Scott, *Sextual Intercourse: Considering Social–Cognitive Predictors and Subsequent Outcomes of Sexting Behavior in Adulthood.* Archives of Sexual Behavior, 2019. **48**(8): p. 2367-2379.

81. Casas, J.A., et al., *Exploring which factors contribute to teens' participation in sexting.* Computers in Human Behavior, 2019. **100**: p. 60-69.

82. Choi, H.J., et al., *Adolescent Sexting Involvement Over 4 Years and Associations With Sexual Activity.* Journal of Adolescent Health, 2019. **65**(6): p. 738-744.

83. Currin, J.M. and R.D. Hubach, *Motivations for Nonuniversity-Based Adults Who Sext Their Relationship Partners.* Journal of Sex and Marital Therapy, 2019. **45**(4): p. 317-327.

84. Clancy, E.M., B. Klettke, and D.J. Hallford, *The dark side of sexting – Factors predicting the dissemination of sexts.* Computers in Human Behavior, 2019. **92**: p. 266-272.

85. Dönmez, Y.E. and N. Soylu, *Online sexual solicitation in adolescents; socio-demographic risk factors and association with psychiatric disorders, especially posttraumatic stress disorder.* Journal of psychiatric research, 2019. **117**: p. 68-73.

86. Falconer, T. and T.P. Humphreys, *Sexting outside the primary relationship: Prevalence, relationship influences, physical engagement, and perceptions of "cheating"...45th Annual Meeting of the Canadian Sex Research Forum, Toronto, Ontario, October 11–13, 2018.* Canadian Journal of Human Sexuality, 2019. **28**(2): p. 134-142.

87. Festl, R., F. Reer, and T. Quandt, *Online sexual engagement and psychosocial well-being: The mediating role of sexual victimization experiences.* Computers in Human Behavior, 2019. **98**: p. 102-110.

88. Florimbio, A.R., et al., *An Examination of Sexting, Sexual Violence, and Alcohol Use Among Men Arrested for Domestic Violence.* Archives of Sexual Behavior, 2019. **48**(8): p. 2381-2387.

89. Gámez-Guadix, M. and E. Mateos-Pérez, *Longitudinal and reciprocal relationships between sexting, online sexual solicitations, and cyberbullying among minors.* Computers in Human Behavior, 2019. **94**: p. 70-76.

90. Gassó, A.M., et al., *Sexting and mental health among a Spanish college sample: An exploratory analysis.* International Journal of Cyber Criminology, 2019. **13**(2): p. 534-547.

91. Ghorashi, Z., *Teenage Sexting and Sexual Behaviors in an Iranian Setting.* Sexuality and Culture, 2019. **23**(4): p. 1274-1282.

92. Harder, S.K., et al., *Digital sexual violence: Image-based sexual abuse among Danish youth*, in *Rape in the Nordic Countries (Open Access)*. 2019. p. 205-223.

93. Ingram, L.A., et al., *Sexual Behaviors, Mobile Technology Use, and Sexting Among College Students in the American South.* American Journal of Health Promotion, 2019. **33**(1): p. 87-96.

94. Jeanfreau, M.M., L. Wright, and K. Noguchi, *Marital Satisfaction and Sexting Behavior Among Individuals in Relationships.* Family Journal, 2019. **27**(1): p. 17-21.

95. Klein, J.L. and D.T. Cooper, *Deviant Cyber-Sexual Activities in Young Adults: Exploring Prevalence and Predictions Using In-Person Sexual Activities and Social Learning Theory.* Archives of Sexual Behavior, 2019. **48**(2): p. 619-630.

96. Klettke, B., et al., *Sexting and Psychological Distress: The Role of Unwanted and Coerced Sexts.* Cyberpsychology, Behavior, and Social Networking, 2019. **22**(4): p. 237-242.

97. Maas, M.K., B.C. Bray, and J.G. Noll, *Online Sexual Experiences Predict Subsequent Sexual Health and Victimization Outcomes Among Female Adolescents: A Latent Class Analysis.* J Youth Adolesc, 2019. **48**(5): p. 837-849.

98. Marengo, D., M. Settanni, and C. Longobardi, *The associations between sex drive, sexual self-concept, sexual orientation, and exposure to online victimization in Italian adolescents: Investigating the mediating role of verbal and visual sexting behaviors.* Children and Youth Services Review, 2019. **102**: p. 18-26.

99. Ojeda, M., R. Del Rey, and S.C. Hunter, *Longitudinal relationships between sexting and involvement in both bullying and cyberbullying.* Journal of Adolescence, 2019. **77**: p. 81-89.

100. Penado, M., et al., *Construction and validation of the intimate images diffusion scale among adolescents.* Frontiers in Psychology, 2019. **10**(JUN).

101. Powell, A. and N. Henry, *Technology-Facilitated Sexual Violence Victimization: Results From an Online Survey of Australian Adults.* J Interpers Violence, 2019. **34**(17): p. 3637-3665.

102. Powell, A., et al., *Image-based sexual abuse: The extent, nature, and predictors of perpetration in a community sample of Australian residents.* Computers in human behavior, 2019. **92**: p. 393-402.

103. Rey, R.D., et al., *Sexting among adolescents: The emotional impact and influence of the need for popularity.* Frontiers in Psychology, 2019. **10**(AUG).

104. Reed, E., et al., *Cyber Sexual Harassment: Prevalence and association with substance use, poor mental health, and STI history among sexually active adolescent girls.* J Adolesc, 2019. **75**: p. 53-62.

105. Ross, J.M., M. Drouin, and A. Coupe, *Sexting Coercion as a Component of Intimate Partner Polyvictimization.* Journal of interpersonal violence, 2019. **34**(11): p. 2269-2291.

106. Tang, W.Y., F. Reer, and T. Quandt, *Investigating sexual harassment in online video games: How personality and context factors are related to toxic sexual behaviors against fellow players.* Aggressive Behavior, 2020. **46**(1): p. 127-135.

107. Titchen, K.E., et al., *Sexting and Young Adolescents: Associations with Sexual Abuse and Intimate Partner Violence.* Journal of Pediatric and Adolescent Gynecology, 2019. **32**(5): p. 481-486.

108. Yépez-Tito, P., M. Ferragut, and M.J. Blanca, *Prevalence and profile of sexting among adolescents in Ecuador.* Journal of Youth Studies, 2019. **22**(4): p. 505-519.

109. Baiden, F., J. Amankwah, and A. Owusu, *Sexting among high school students in a metropolis in Ghana: an exploratory and descriptive study.* Journal of Children and Media, 2020. **14**(3): p. 361-375.

110. Calvete, E., et al., *Internet-risk classes of adolescents, dispositional mindfulness and health-related quality of life: A mediational model.* Cyberpsychology, Behavior, and Social Networking, 2020. **23**(8): p. 533-540.

111. Clancy, E.M., et al., *Sharing is not always caring: Understanding motivations and behavioural associations with sext dissemination.* Computers in Human Behavior, 2020. **112**.

112. Currin, J.M., et al., *Sextually aroused: A mixed-methods analysis of how it feels for romantic and sexual partners to send and receive sext messages.* Computers in Human Behavior, 2020. **113**.

113. Corcoran, C.T., et al., *The Development and Piloting of a Digital Checklist to Increase Access and Usage of Campus Online Sexual Violence Resources.* Health Education and Behavior, 2020. **47**(1_suppl): p. 36S-43S.

114. Confalonieri, E., et al., *What are you sexting? Parental practices, sexting attitudes and behaviors among Italian adolescents.* BMC Psychology, 2020. **8**(1).

115. Cornelius, T.L., et al., *Consensual sexting among college students: The interplay of coercion and intimate partner aggression in perceived consequences of sexting.* International Journal of Environmental Research and Public Health, 2020. **17**(19): p. 1-18.

116. Cavalcanti, J.G., et al., *Psychometric Properties of the Cyber Dating Abuse Questionnaire.* Psico usf, 2020. **25**(2): p. 285-296.

117. de Abreu Silva, T.E., R. de Godoy Pereira, and D.A. Baltieri, *Empathy and sexual impulsiveness among medical students who admit to sexting partners' intimate images.* Journal of Human Growth and Development, 2020. **30**(1): p. 111-119.

118. Dolev-Cohen, M. and T. Ricon, *Demystifying sexting: Adolescent sexting and its associations with parenting styles and sense of parental social control in Israel.* Cyberpsychology, 2020. **14**(1).

119. Dodaj, A., K. Sesar, and S. Jerinić, *A Prospective Study of High-School Adolescent Sexting Behavior and Psychological Distress.* Journal of Psychology: Interdisciplinary and Applied, 2020. **154**(2): p. 111-128.

120. Florimbio, A.R., et al., *Sexting and Substance Use among Women Arrested for Domestic Violence: A Brief Report.* Substance Abuse: Research & Treatment, 2020. **14**: p. 1-3.

121. Gassó, A.M., K. Mueller‐johnson, and I. Montiel, *Sexting, online sexual victimization, and psychopathology correlates by sex: Depression, anxiety, and global psychopathology.* International Journal of Environmental Research and Public Health, 2020. **17**(3).

122. Geeng, C., J. Hutson, and F. Roesner. *Usable sexurity: Studying people's concerns and strategies when sexting*. in *Proceedings of the 16th Symposium on Usable Privacy and Security, SOUPS 2020*. 2020.

123. Gil-Llario, M.D., et al., *The phenomenon of sexting among spanish teenagers: Prevalence, attitudes, motivations and explanatory variables.* Anales de Psicologia, 2020. **36**(2): p. 210-219.

124. Gil-Llario, M.D., et al., *Culture as an influence on sexting attitudes and behaviors: A differential analysis comparing adolescents from Spain and Colombia.* International Journal of Intercultural Relations, 2020. **79**: p. 145-154.

125. Gómez-García, G., et al., *Sexting among university students: Links to internet addiction and psychological variables.* Journal of Drug and Alcohol Research, 2020. **9**(6).

126. Kim, S., et al., *Prevalence and Correlates of Sexting Behaviors in a Provincially Representative Sample of Adolescents.* Canadian Journal of Psychiatry, 2020. **65**(6): p. 401-408.

127. Lucić, M., V. Baćak, and A. Štulhofer, *The role of peer networks in adolescent pornography use and sexting in Croatia.* Journal of Children and Media, 2020. **14**(1): p. 110-127.

128. Molla-Esparza, C., J.M. Losilla, and E. López-González, *Prevalence of sending, receiving and forwarding sexts among youths: A three-level meta-analysis.* PLoS ONE, 2020. **15**(12 December).

129. Morelli, M., et al., *The role of HEXACO personality traits in different kinds of sexting:A cross-cultural study in 10 countries.* Computers in Human Behavior, 2020. **113**.

130. Oswald, F., et al., *I'll Show You Mine so You'll Show Me Yours: Motivations and Personality Variables in Photographic Exhibitionism.* The Journal of sex research, 2020. **57**(5): p. 597-609.

131. Paquette, S. and F. Cortoni, *The Development and Validation of the Cognitions of Internet Sexual Offending (C-ISO) Scale.* Sex Abuse, 2020. **32**(8): p. 907-930.

132. Reed, L.A., et al., *How do adolescents experience sexting in dating relationships? Motivations to sext and responses to sexting requests from dating partners.* Children and Youth Services Review, 2020. **109**.

133. Snaychuk, L.A. and M.L. O’Neill, *Technology-Facilitated Sexual Violence: Prevalence, Risk, and Resiliency in Undergraduate Students.* Journal of Aggression, Maltreatment and Trauma, 2020. **29**(8): p. 984-999.

134. McKinlay, T. and T. Lavis, *Why did she send it in the first place? Victim blame in the context of ‘revenge porn’.* Psychiatry, Psychology and Law, 2020. **27**(3): p. 386-396.

135. Muncaster, L. and I. Ohlsson, *Sexting: predictive and protective factors for its perpetration and victimisation.* Journal of Sexual Aggression, 2020. **26**(3): p. 346-358.

136. Niehuis, S., et al., *Guilty pleasure? Communicating sexually explicit content on dating apps and disillusionment with app usage.* Human Communication Research, 2020. **46**(1): p. 55-85.

137. Turban, J.L., et al., *Posting Sexually Explicit Images or Videos of Oneself Online Is Associated With Impulsivity and Hypersexuality but Not Measures of Psychopathology in a Sample of US Veterans.* Journal of Sexual Medicine, 2020. **17**(1): p. 163-167.

138. Yépez-Tito, P., M. Ferragut, and M.J. Blanca, *Sexting in adolescence: The use of technology and parental supervision.* Revista Latinoamericana de Psicologia, 2020. **52**(1): p. 115-130.

139. Yu, K. and Y. Zheng, *Sexting and emotional reactions to hooking up among Chinese college students: Moderated mediation effects of loneliness and number of hookup partners.* Personality and Individual Differences, 2020. **167**.

140. Burić, J., J.R. Garcia, and A. Štulhofer, *Is sexting bad for adolescent girls’ psychological well-being? A longitudinal assessment in middle to late adolescence.* New Media and Society, 2021. **23**(7): p. 2052-2071.

141. Brown, C. and K. Hegarty, *Development and validation of the TAR Scale: A measure of technology-facilitated abuse in relationships.* Computers in human behavior reports, 2021. **3**: p. 100059.

142. Del Rey, R., M. Ojeda, and J.A. Casas, *Validation of the sexting behavior and motives questionnaire (Sbm-q).* Psicothema, 2021. **33**(2): p. 287-295.

143. Gámez-Guadix, M., et al., *Unraveling cyber sexual abuse of minors: Psychometrics properties of the Multidimensional Online Grooming Questionnaire and prevalence by sex and age.* Child Abuse Negl, 2021. **120**: p. 105250.

144. Gámez-Guadix, M. and D. Incera, *Homophobia is online: Sexual victimization and risks on the internet and mental health among bisexual, homosexual, pansexual, asexual, and queer adolescents.* Computers in Human Behavior, 2021. **119**.

145. Gil-Llario, M.D., et al., *Analysis of demographic, psychological and cultural aspects associated with the practice of sexting in Mexican and Spanish adolescents.* International Journal of Intercultural Relations, 2021. **82**: p. 197-206.

146. García-González, A., et al., *The Phenomenon of Sexting Among Mexican and Spanish University Students: A Multigroup Model.* Sexuality and Culture, 2021. **25**(3): p. 939-959.

147. Hinduja, S. and J.W. Patchin, *Digital Dating Abuse Among a National Sample of U.S. Youth.* Journal of Interpersonal Violence, 2021. **36**(23-24): p. 11088-11108.

148. López-Cepero, J., J. Vallejos-Saldarriaga, and M. Merino-García, *Digital Intimate Partner Violence Among Peruvian Youths: Validation of an Instrument and a Theoretical Proposal.* J Interpers Violence, 2021. **36**(11-12): p. 5167-5185.

149. Barroso, R., et al., *Abusive Sexting in Adolescence: Prevalence and Characteristics of Abusers and Victims.* Frontiers in Psychology, 2021. **12**.

150. Boer, S., et al., *Prevalence and Correlates of Sext-Sharing Among a Representative Sample of Youth in the Netherlands.* Frontiers in Psychology, 2021. **12**.

151. Beckmeyer, J.J., D. Herbenick, and H. Eastman-Mueller, *Sexting with Romantic Partners During College: Who Does It, Who Doesn’t and Who Wants To.* Sexuality and Culture, 2022. **26**(1): p. 48-66.

152. Bianchi, D., et al., *A Bad Romance: Sexting Motivations and Teen Dating Violence.* Journal of Interpersonal Violence, 2021. **36**(13-14): p. 6029-6049.

153. Bianchi, D., et al., *Patterns of love and sexting in teen dating relationships: The moderating role of conflicts.* New Directions for Child and Adolescent Development, 2021. **2021**(178): p. 133-155.

154. Bianchi, D., et al., *Individual differences and developmental trends in sexting motivations.* Current psychology (New Brunswick, N.J.), 2021. **40**(9): p. 4531-4540.

155. Brown, C., L. Sanci, and K. Hegarty, *Technology-facilitated abuse in relationships: Victimisation patterns and impact in young people.* Computers in human behavior, 2021. **124**: p. 106897.

156. Calvete, E., et al., *Moderating factors of the association between being sexually solicited by adults and active online sexual behaviors in adolescents.* Computers in Human Behavior, 2021. **124**.

157. Champion, A., F. Oswald, and C.L. Pedersen, *Technology-facilitated sexual violence and suicide risk: A serial mediation model investigating bullying, depression, perceived burdensomeness, and thwarted belongingness.* The Canadian journal of human sexuality, 2021. **30**(1): p. 125-141.

158. Clancy, E.M., et al., *Sext dissemination: Differences across nations in motivations and associations.* International Journal of Environmental Research and Public Health, 2021. **18**(5): p. 1-16.

159. Clancy, E.M., et al., *Just Checking It Out? Motivations for and Behavioral Associations With Visiting “Slutpages” in the United States and Australia.* Frontiers in Psychology, 2021. **12**.

160. Copp, J.E., E.A. Mumford, and B.G. Taylor, *Online sexual harassment and cyberbullying in a nationally representative sample of teens: Prevalence, predictors, and consequences.* Journal of Adolescence, 2021. **93**: p. 202-211.

161. Courtice, E.L., et al., *Unsolicited Pics and Sexual Scripts: Gender and Relationship Context of Compliant and Non-consensual Technology-Mediated Sexual Interactions.* Frontiers in Psychology, 2021. **12**.

162. Durmuş, H. and Y. Solak, *Sexting behavior among adults in Turkey and its relationship with self-esteem and risky sexual behavior.* Sexual and Relationship Therapy, 2021.

163. Foody, M., et al., *“It's not just sexy pics”: An investigation into sexting behaviour and behavioural problems in adolescents.* Computers in Human Behavior, 2021. **117**.

164. Gassó, A.M. and E. Gómez-Durán, *Psychopathological profile of sexting coercion perpetrators.* Revista Espanola de Medicina Legal, 2021. **47**(4): p. 157-163.

165. Gassó, A.M., et al., *Mental health correlates of sexting coercion perpetration and victimisation in university students by gender.* Journal of Sexual Aggression, 2021. **27**(2): p. 247-263.

166. Gassó, A.M., K. Mueller-Johnson, and E.L. Gómez-Durán, *Victimization as a result of non-consensual dissemination of sexting and psychopathology correlates: An exploratory analysis.* International Journal of Environmental Research and Public Health, 2021. **18**(12).

167. Hernández, M.P., et al., *The risk of sexual-erotic online behavior in adolescents – Which personality factors predict sexting and grooming victimization?* Computers in Human Behavior, 2021. **114**.

168. Howard, D., et al., *Body image self-consciousness and sexting among heterosexual and non-exclusively heterosexual individuals.* New Media and Society, 2021. **23**(5): p. 1217-1235.

169. Hunter, S.C., et al., *A Social-Ecological Approach to Understanding Adolescent Sexting Behavior.* Archives of Sexual Behavior, 2021. **50**(6): p. 2347-2357.

170. Kafaee, N. and T. Kohut, *Online sexual experiences and relationship functioning in long distance relationships.* Canadian Journal of Human Sexuality, 2021. **30**(1): p. 15-25.

171. Longobardi, C., et al., *The Role of Body Image Concerns in Online Sexual Victimization among Female Adolescents: The Mediating Effect of Risky Online Behaviors.* Journal of Child and Adolescent Trauma, 2021. **14**(1): p. 51-60.

172. Lu, Y., E. Baumler, and J.R. Temple, *Multiple forms of sexting and associations with psychosocial health in early adolescents.* International Journal of Environmental Research and Public Health, 2021. **18**(5): p. 1-7.

173. Maas, M.K., et al., *Slutpage Use Among U.S. College Students: The Secret and Social Platforms of Image-Based Sexual Abuse.* Archives of Sexual Behavior, 2021. **50**(5): p. 2203-2214.

174. Matotek, L., et al., *Sexting and Relationship Satisfaction Among Emerging Adults: Dispelling the Myth.* Sexuality and Culture, 2021. **25**(4): p. 1192-1203.

175. Mori, C., et al., *Patterns of sexting and sexual behaviors in youth: A Latent Class Analysis.* Journal of Adolescence, 2021. **88**: p. 97-106.

176. Morelli, M., et al., *The relationship between dark triad personality traits and sexting behaviors among adolescents and young adults across 11 countries.* International Journal of Environmental Research and Public Health, 2021. **18**(5): p. 1-25.

177. Reer, F., R. Wendt, and T. Quandt, *A Longitudinal Study on Online Sexual Engagement, Victimization, and Psychosocial Well-Being.* Frontiers in Psychology, 2021. **12**.

178. Resett, S., *Grooming online, sexting and emotional problems in Argentinean adolescents.* Ciencias Psicologicas, 2021. **15**(1).

179. Rodríguez-Castro, Y., et al., *Intimate partner cyberstalking, sexism, pornography and sexting in adolescents: New challenges for sex education.* International Journal of Environmental Research and Public Health, 2021. **18**(4): p. 1-15.

180. Ståhl, S. and I. Dennhag, *Online and offline sexual harassment associations of anxiety and depression in an adolescent sample.* Nord J Psychiatry, 2021. **75**(5): p. 330-335.

181. Van Ouytsel, J., et al., *Sexting, pressured sexting and associations with dating violence among early adolescents.* Computers in Human Behavior, 2021. **125**.

182. Van Ouytsel, J., et al., *Sexting, pressured sexting and image-based sexual abuse among a weighted-sample of heterosexual and LGB-youth.* Computers in Human Behavior, 2021. **117**.

183. Yépez-Tito, P., M. Ferragut, and M.J. Blanca, *Character strengths as protective factors against engagement in sexting in adolescence.* Anales de Psicologia, 2021. **37**(1): p. 142-148.

184. Walker, K., et al., *Nonconsensual Sharing of Private Sexually Explicit Media Among University Students.* Journal of Interpersonal Violence, 2021. **36**(17-18): p. NP9078-NP9108.

185. Wachs, S., et al., *How are consensual, non-consensual, and pressured sexting linked to depression and self-harm? The moderating effects of demographic variables.* International Journal of Environmental Research and Public Health, 2021. **18**(5): p. 1-16.

186. Widman, L., et al., *Sexual Communication in the Digital Age: Adolescent Sexual Communication with Parents and Friends About Sexting, Pornography, and Starting Relationships Online.* Sexuality and Culture, 2021. **25**(6): p. 2092-2109.

187. Buchanan, N. and A. Mahoney, *Development of a scale measuring online sexual harassment: Examining gender differences and the emotional impact of sexual harassment victimization online.* Legal and Criminological Psychology, 2022. **27**(1): p. 63-81.

188. Belotti, F., et al., *Towards ‘romantic media ideologies’: digital dating abuse seen through the lens of social media and/or dating in teenage narratives.* Communication Review, 2022. **25**(1): p. 30-53.

189. Champion, A.R., et al., *Examining the Gendered Impacts of Technology-Facilitated Sexual Violence: A Mixed Methods Approach.* Archives of Sexual Behavior, 2022. **51**(3): p. 1607-1624.

190. Calvete, E., I. Orue, and M. Gámez-Guadix, *A Preventive Intervention to Reduce Risk of Online Grooming Among Adolescents.* Psychosocial Intervention, 2022. **31**(3): p. 177-184.

191. Califano, G., M. Capasso, and D. Caso, *Exploring the roles of online moral disengagement, body esteem, and psychosexual variables in predicting sexting motivations and behaviours.* Computers in Human Behavior, 2022. **129**.

192. Caponnetto, P., F.F. Gervasi, and M. Maglia, *Sexting: a potential addiction or an adaptive behavior to COVID-19 social distancing and stay-at-home policies? A qualitative study.* Journal of Addictive Diseases, 2022. **40**(1): p. 84-91.

193. Currin, J.M., *Linking Sexting Expectancies with Motivations to Sext.* European Journal of Investigation in Health, Psychology and Education, 2022. **12**(2): p. 209-217.

194. Currin, J.M., B.L. Golden, and R.D. Hubach, *Predicting type of sext message sent in adults 25 and older using motivations to sext and relational attachment.* Current Psychology, 2022. **41**(3): p. 1526-1533.

195. Díaz-Aguado, M.J. and R. Martínez-Arias, *Types of Male Adolescent Violence Against Women in Three Contexts: Dating Violence Offline, Dating Violence Online, and Sexual Harassment Online Outside a Relationship.* Frontiers in Psychology, 2022. **13**.

196. Dodaj, A., et al., *Through the Eyes of Young People: A Qualitative Study of Sexting Among Croatian and Bosnian and Herzegovinian College Students.* Sexuality and Culture, 2022. **26**(5): p. 1885-1918.

197. Dolev-Cohen, M. and T. Ricon, *Dysfunctional Parent–Child Communication About Sexting During Adolescence.* Archives of Sexual Behavior, 2022. **51**(3): p. 1689-1702.

198. Dolev-Cohen, M. and T. Ricon, *Talking About Sexting: Association Between Parental Factors and Quality of Communication About Sexting With Adolescent Children in Jewish and Arab Society in Israel.* Journal of Sex and Marital Therapy, 2022. **48**(5): p. 429-443.

199. Dolev-Cohen, M. and T. Ricon, *Parent-child dysfunctional communication about sexting: The role of parental characteristics and parental mediation.* Annual Review of CyberTherapy and Telemedicine, 2022. **20**: p. 97-101.

200. Fissel, E.R., et al., *A New Frontier: The Development and Validation of the Intimate Partner Cyber Abuse Instrument.* Social science computer review, 2022. **40**(4): p. 974-993.

201. Jeanfreau, M.M., et al., *Personality and Sexting: The Relationship Between Sexting Behaviors, Sexting Expectations, and the Big Five.* Family Journal, 2022. **30**(1): p. 50-58.

202. Kokkinos, C.M. and C. Krommida, *Prevalence of Sexting among Greek University Students: A Matter of Relationships?* The journal of psychology, 2022. **156**(7): p. 459-477.

203. Gámez-Guadix, M., et al., *Assessing image-based sexual abuse: Measurement, prevalence, and temporal stability of sextortion and nonconsensual sexting ("revenge porn") among adolescents.* J Adolesc, 2022. **94**(5): p. 789-799.

204. Pasca, P., et al., *Detecting online grooming at its earliest stages: development and validation of the Online Grooming Risk Scale.* Mediterranean Journal of Clinical Psychology, 2022. **10**(1).

205. Qu, J., et al., *Sexting Victimization Among Dating App Users: A Comparison of U.S. and Chinese College Students.* Journal of Interpersonal Violence, 2022. **37**(19-20): p. NP17109-NP17132.

206. Resett, S., P.G. Caino, and B. Mesurado, *Emotional Problems, Dark Personality, Sexting and Grooming in Adolescents: the role of Gender and Age.* Revista CES Psicologia, 2022. **15**(2): p. 24-43.

207. Romero-Rodríguez, J.M., et al., *Sexting in times of confinement. An analysis of sending online sexual content before and during COVID-19 pandemic amongst university students.* KOME, 2022. **10**(1): p. 19-31.

208. Sparks, B., *A Snapshot of Image-Based Sexual Abuse (IBSA): Narrating a Way Forward.* Sexuality Research and Social Policy, 2022. **19**(2): p. 689-704.

209. Zagloul, N.M., et al., *Technology facilitated sexual violence: a comparative study between working and non-working females in Egypt before and during the COVID-19 pandemic.* Egyptian Journal of Forensic Sciences, 2022. **12**(1).

210. Barrense-Dias, Y., et al., *Sending One's Own Intimate Image: Sexting Among Middle-School Teens.* Journal of School Health, 2022. **92**(4): p. 353-360.

211. Bielčiková, K. and K. Hollá, *Media education as a means of sexting prevention.* Journal of Education Culture and Society, 2022. **13**(1): p. 111-125.

212. Bragard, E. and C.B. Fisher, *Associations between sexting motivations and consequences among adolescent girls.* Journal of adolescence, 2022. **94**(1): p. 5-18.

213. Burén, J., K. Holmqvist Gattario, and C. Lunde, *What Do Peers Think About Sexting? Adolescents’ Views of the Norms Guiding Sexting Behavior.* Journal of Adolescent Research, 2022. **37**(2): p. 221-249.

214. Corcoran, E., et al., *Youth sexting and associations with parental media mediation.* Computers in Human Behavior, 2022. **132**.

215. Crapolicchio, E., et al., *Sexting and the experience of non-consensual dissemination of sexts. The moderator role of self-objectification.* Psicologia Sociale, 2022. **17**(2): p. 207-224.

216. Dardis, C.M. and E.C. Richards, *Nonconsensual Distribution of Sexually Explicit Images Within a Context of Coercive Control: Frequency, Characteristics, and Associations with Other Forms of Victimization.* Violence Against Women, 2022. **28**(15-16): p. 3933-3954.

217. Finkelhor, D., H. Turner, and D. Colburn, *Prevalence of Online Sexual Offenses Against Children in the US.* JAMA Network Open, 2022. **5**(10): p. E2234471.

218. Garner, A.R., et al., *Negative urgency moderates the association between compulsive sexual behaviors and sending unsolicited sexts among men in residential treatment for substance use disorders.* Computers in Human Behavior, 2022. **136**.

219. Greer, K.M., et al., *Differences Between Gender and Relationship Status in Motivations and Consequences of Consensual Sexting Among Emerging Adults.* Sexuality and Culture, 2022. **26**(4): p. 1432-1451.

220. Gargano, M., et al., *Sexting in adolescence: what are the reasons? An empirical study.* Clinica Terapeutica, 2022. **173**(5): p. 407-413.

221. Guest, C. and A. Denes, *Too much too soon?: Perceived appropriateness of sexting across stages of relationship development and attachment tendencies among emerging adults.* Computers in Human Behavior, 2022. **137**.

222. Huiskes, P., M.A.P. Dinis, and S. Caridade, *Technology-Facilitated Sexual Violence Victimization during the COVID-19 Pandemic: Behaviors and Attitudes.* Journal of Aggression, Maltreatment and Trauma, 2022. **31**(9): p. 1148-1167.

223. Kamar, E., et al., *Parental guardianship and online sexual grooming of teenagers: A honeypot experiment.* Computers in Human Behavior, 2022. **137**.

224. Maes, C. and L. Vandenbosch, *Physically distant, virtually close: Adolescents’ sexting behaviors during a strict lockdown period of the COVID-19 pandemic.* Computers in Human Behavior, 2022. **126**.

225. Marcum, C.D., B.H. Zaitzow, and G.E. Higgins, *The role of sexting and related behaviors to victimization via nonconsensual pornography: an exploratory analysisof university students.* Journal of Aggression, Conflict and Peace Research, 2022. **14**(1): p. 43-60.

226. Ng, M.Y., et al., *Sexting and behavioral health in first-time justice-involved adolescents.* Children and Youth Services Review, 2022. **132**.

227. Noorishad, P.G. and D. Trottier, *Investigating the relationship between sexting and sexual coercion.* Sexologies, 2022. **31**(1): p. e8-e15.

228. Powell, A., et al., *Perpetration of Image-Based Sexual Abuse: Extent, Nature and Correlates in a Multi-Country Sample.* Journal of Interpersonal Violence, 2022. **37**(23-24): p. NP22864-NP22889.

229. Powell, A., et al., *A multi-country study of image-based sexual abuse: extent, relational nature and correlates of victimisation experiences.* Journal of Sexual Aggression, 2022.

230. Paquette, M.M., et al., *Heterosexual, Cisgender and Gender and Sexually Diverse Adolescents’ Sexting Behaviors: The Role of Body Appreciation.* Journal of Youth and Adolescence, 2022. **51**(2): p. 278-290.

231. Rollero, C. and S. Pagliaro, *Moral foundations and victim blaming in case of non-consensual dissemination of one's sexual images: A preliminary study.* Psicologia Sociale, 2022. **17**(2): p. 195-205.

232. Durán, M. and C. Rodríguez-Domínguez, *Sending of Unwanted Dick Pics as a Modality of Sexual Cyber-Violence: An Exploratory Study of Its Emotional Impact and Reactions in Women.* Journal of Interpersonal Violence, 2023. **38**(5-6): p. 5236-5261.

233. Harper, C.A., et al., *Development and Validation of the Beliefs About Revenge Pornography Questionnaire.* Sex Abuse, 2023. **35**(6): p. 748-783.

234. Hussain, S., et al., *Why women avoid sexting: Mediating role of depression and guilt.* Current Psychology, 2023. **42**(17): p. 14132-14146.

235. Bianchi, D., et al., *Love in Quarantine: Sexting, Stress, and Coping During the COVID-19 Lockdown.* Sexuality Research and Social Policy, 2023. **20**(2): p. 465-478.

236. Beckmeyer, J.J., *Sexting and the Dynamics of Emerging Adults’ Romantic Partnerships.* Sexuality and Culture, 2023.

237. Bogner, J., et al., *Sexting as a Predictor of First-Time Sexual Behavior Among At-Risk Early Adolescents.* Journal of Early Adolescence, 2023. **43**(4): p. 516-538.

238. Bonfanti, R.C., et al., *The role of sexting in couple wellbeing for Italian women during the second wave of the COVID-19 pandemic.* Frontiers in Psychology, 2023. **14**.

239. Borgogna, N.C., et al., *Further Understanding the Correlations Between Sexting and Mental Health: Considerations for Sex and Sexual Identity.* Cyberpsychology, Behavior, and Social Networking, 2023. **26**(5): p. 323-331.

240. Brighi, A., et al., *Prevalence and risk factors for nonconsensual distribution of intimate images among Italian young adults: Implications for prevention and intervention.* International Journal of Clinical and Health Psychology, 2023. **23**(4).

241. Cross, C., K. Holt, and R.L. O’Malley, *“If U Don’t Pay they will Share the Pics”: Exploring Sextortion in the Context of Romance Fraud.* Victims and Offenders, 2023. **18**(7): p. 1194-1215.

242. Currin, J.M., A.E. Evans, and S. Garos, *In a relationship, who tends to sext first?* Sexual and Relationship Therapy, 2023. **38**(4): p. 586-602.

243. Chan, L.S. and B. Wu-Ouyang, *Sexting Among Men Who Have Sex with Men in Hong Kong and Taiwan: Roles of Sensation-Seeking, Gay Identity, and Muscularity Ideal.* Archives of Sexual Behavior, 2023. **52**(6): p. 2373-2384.

244. Checkalski, O.R., S.J. Gervais, and K.J. Holland, *A triangulation study of young Women's motivations for sending nudes to men.* Computers in Human Behavior, 2023. **140**.

245. Choi, J., et al., *The Relationship of Risky Online Behaviors and Adverse Childhood Experiences to Online Sexual Victimization Among Korean Female Adolescents.* J Interpers Violence, 2023. **38**(3-4): p. 3637-3660.

246. Cuccì, G., et al., *Risk or fun? Adolescent attitude towards sexting and parental practices.* Journal of Family Studies, 2023.

247. Dodaj, A. and K. Sesar, *Individual and Cross-Cultural Predictors of Sexting Among Adults from Bosnia and Herzegovina and Croatia.* Sexuality Research and Social Policy, 2023. **20**(4): p. 1537-1551.

248. Dodaj, A. and K. Sesar, *Sexting coercion within romantic context: a test of Akers’ social learning theory.* Journal of Sexual Aggression, 2023.

249. Dodaj, A., et al., *Using Vignettes In Qualitative Research To Assess Young Adults’ Perspectives Of Sexting Behaviours.* Human Technology, 2023. **19**(1): p. 103-120.

250. Dolev-Cohen, M., *The association between sexting motives and behavior as a function of parental and peers' role.* Computers in Human Behavior, 2023. **147**.

251. Eaton, A.A., D. Ramjee, and J.F. Saunders, *The Relationship between Sextortion during COVID-19 and Pre-pandemic Intimate Partner Violence: A Large Study of Victimization among Diverse U.S Men and Women.* Victims and Offenders, 2023. **18**(2): p. 338-355.

252. Finkelhor, D., H. Turner, and D. Colburn, *Which dynamics make online child sexual abuse and cyberstalking more emotionally impactful: Perpetrator identity and images?* Child Abuse Negl, 2023. **137**: p. 106020.

253. Falconer, T.A., et al., *Influences on sexting in an intimate relationship: Motivations, risks, communication, personality traits, and relationship variables.* Canadian Journal of Human Sexuality, 2023. **32**(3): p. 275-288.

254. Flynn, A., et al., *Victim-blaming and image-based sexual abuse.* Journal of Criminology, 2023. **56**(1): p. 7-25.

255. Foody, M., et al., *Sexting behaviour among adolescents: Do friendship quality and social competence matter?* Computers in Human Behavior, 2023. **142**.

256. Garrido-Macías, M., et al., *Sexting is not always wanted: consequences on satisfaction and the role of sexual coercion and online sexual victimization.* Anales de Psicologia, 2023. **39**(3): p. 354-363.

257. Goh, Y.S., S.A. Tan, and S.W. Gan, *Sexting motives and sexting behavior among emerging adults in Malaysia during the COVID-19 pandemic lockdown.* Gender, Technology and Development, 2023. **27**(1): p. 136-156.

258. Gesselman, A.N., et al., *Engagement with Emerging Forms of Sextech: Demographic Correlates from a National Sample of Adults in the United States.* Journal of Sex Research, 2023. **60**(2): p. 177-189.

259. Hassan, H., et al., *Personality Traits, Self-Control, and Sexting Attitudes Among Young Adults.* Pakistan Journal of Psychological Research, 2023. **38**(1): p. 147-164.

260. Holfeld, B., et al., *A Latent Profile Analysis of the Consensual and Non-Consensual Sexting Experiences among Canadian Adolescents.* Youth and Society, 2023.

261. Hong, J.S., et al., *Pathways from Polyvictimization to Offline and Online Sexual Harassment Victimization Among South Korean Adolescents.* Arch Sex Behav, 2023. **52**(7): p. 2779-2788.

262. Huntington, C. and G. Rhoades, *Associations of sexting with dating partners with adolescents’ romantic relationship behaviors and attitudes.* Sexual and Relationship Therapy, 2023. **38**(4): p. 780-795.

263. Huang, T.F., et al., *Adolescent Use of Dating Applications and the Associations with Online Victimization and Psychological Distress.* Behavioral Sciences, 2023. **13**(11).

264. Kamar, E., et al., *The Relevance of Targets’ Sexual Knowledge in the Progression of Online Sexual Grooming Events: Findings from an Online Field Experiment.* Justice Quarterly, 2023.

265. Karasavva, V., et al., *From myth to reality: sexual image abuse myth acceptance, the Dark Tetrad, and non-consensual intimate image dissemination proclivity.* Journal of Sexual Aggression, 2023. **29**(1): p. 51-67.

266. Kokkinos, C.M., et al., *A Latent Profile Analysis of Greek University Students’ Sexting Profiles: Associations with Big Five Personality Traits.* Archives of Sexual Behavior, 2023.

267. Lunde, C., et al., *Sexting experiences and motivations among adolescents with ADHD and ASD.* Computers in Human Behavior, 2023. **140**.

268. Maes, C., J. Van Ouytsel, and L. Vandenbosch, *Victim Blaming and Non-Consensual Forwarding of Sexts Among Late Adolescents and Young Adults.* Archives of Sexual Behavior, 2023. **52**(4): p. 1767-1783.

269. Molla-Esparza, C., J.M. Losilla, and E. López-González, *Sexting prevalence in high-school students depends on the addressee, media format, explicitness of sexts, and gender.* Behaviour and Information Technology, 2023. **42**(16): p. 2852-2870.

270. Molla-Esparza, C., et al., *Sexting Behavior Predictors Vary With Addressee and the Explicitness of the Sexts.* Youth and Society, 2023. **55**(4): p. 749-771.

271. Morelli, M., et al., *The Relationship Between Trait Emotional Intelligence and Sexting in Adolescence.* Sexuality Research and Social Policy, 2023.

272. Morelli, M., et al., *Sexting Behaviors Before and During COVID-19 in Italian and Colombian Young Adults.* Sexuality Research and Social Policy, 2023. **20**(4): p. 1515-1527.

273. Mussap, A.J., E.M. Clancy, and B. Klettke, *Attitudes and Beliefs Associated with Cyberbullying and Non-Consensual Sexting in Cisgender and Transgender Adults.* Gender Issues, 2023. **40**(1): p. 65-85.

274. Mussap, A.J., E.M. Clancy, and B. Klettke, *The Effects of Cyberbullying and Non-consensual Sexting on Gender Minority Stress and Psychological Functioning in Transgender Adults.* Journal of LGBTQ Issues in Counseling, 2023. **17**(3): p. 202-222.

275. Okumu, M., et al., *Digital technologies, equitable gender norms, and sexual health practices across sexting patterns among forcibly displaced adolescents in the slums of Kampala, Uganda.* Computers in Human Behavior, 2023. **138**.

276. Parti, K., C.E. Sanders, and E.K. Englander, *Sexting at an Early Age: Patterns and Poor Health-Related Consequences of Pressured Sexting in Middle and High School*.* Journal of School Health, 2023. **93**(1): p. 73-81.

277. Pedersen, W., et al., *Sexual Victimization in the Digital Age: A Population-Based Study of Physical and Image-Based Sexual Abuse Among Adolescents.* Archives of Sexual Behavior, 2023. **52**(1): p. 399-410.

278. Pistoni, C., et al., *What are the predictors of sexting behavior among adolescents? The positive youth development approach.* Journal of Adolescence, 2023. **95**(4): p. 661-671.

279. Ragona, A., et al., *Motivations, Behaviors and Expectancies of Sexting: The Role of Defensive Strategies and Social Media Addiction in a Sample of Adolescents.* International Journal of Environmental Research and Public Health, 2023. **20**(3).

280. Rollero, C., M. Teresi, and S. Pagliaro, *The Role of Sexting on the Perception of Image-Based Sexual Abuse.* Journal of Interpersonal Violence, 2023. **38**(21-22): p. 11727-11744.

281. Sesar, K., A. Dodaj, and M. Vučić, *The Relationship between Sexting, Risky Sexual Behaviour and Features of Borderline Personality Disorder.* Mediterranean Journal of Clinical Psychology, 2023. **11**(1).

282. Karasavva, V., et al., *Putting the Y in cyberflashing: Exploring the prevalence and predictors of the reasons for sending unsolicited nude or sexual images.* Computers in Human Behavior, 2023. **140**.

283. Ricon, T. and M. Dolev-Cohen, *Sexting Behavior by Young Adults: The Correlation between Emotion Regulation and Moral Judgment.* American Journal of Sexuality Education, 2023.

284. Schokkenbroek, J.M., et al., *Receive, forward, repeat: The link between sexting intention, sexting attitudes, and non-consensual sexting behaviours.* Telematics and Informatics, 2023. **84**.

285. Sparks, B., S. Stephens, and S. Trendell, *Image-based sexual abuse: Victim-perpetrator overlap and risk-related correlates of coerced sexting, non-consensual dissemination of intimate images, and cyberflashing.* Computers in Human Behavior, 2023. **148**.

286. Sciacca, B., et al., *Nonconsensual Dissemination of Sexual Images Among Adolescents: Associations With Depression and Self-Esteem.* Journal of Interpersonal Violence, 2023. **38**(15-16): p. 9438-9464.

287. Catarina Almeida, T. and I. Barreiros, *Online grooming among Portuguese adolescents and the COVID-19 lockdown: Relationship with other types of victimization.* Children and Youth Services Review, 2024. **156**.

288. Cary, K.M., M.K. Maas, and R.P. Bowles, *Development and Validation of the Online Sexual Objectification Experiences Scale Among College Women.* Psychology of women quarterly, 2024. **48**(3): p. 443-459.

289. Damra, J.K., S. Abujilban, and M.M. Akour, *The Cyber Intimate Partner Violence: Prevalence, Context, and Relationship With In-Person Intimate Violence Victimization.* Journal of family issues, 2024. **45**(7): p. 1683-1705.

290. Kowalski, R. and M. Thompson, *Expanding the Sexual Experiences Survey to Include Technology Facilitated Sexual Exploitation.* The Journal of sex research, 2024. **61**(6): p. 897-903.

291. Maes, C., J. Van Ouytsel, and L. Vandenbosch, *Active bystanders in the forwarding of sexting messages: Applying a theory of planned behavior in youth.* New media & society, 2024.

292. Martínez-Bacaicoa, J., M.A. Sorrel, and M. Gámez-Guadix, *Development and Validation of Technology-Facilitated Sexual Violence Perpetration and Victimization Scales Among Adults.* Assessment (Odessa, Fla.), 2024: p. 10731911241229575-10731911241229575.

293. Martínez Soto, A., et al., *Cyber dating abuse in adolescents: Myths of romantic love, sexting practices and bullying.* Computers in Human Behavior, 2024. **150**.

294. Muñoz-Fernández, N. and V. Sánchez-Jiménez, *Intimate partner violence among LGB and heterosexual adults: Prevalence and associated minority stress factors.* Sexual and Gender Diversity in Social Services, 2025. **37**(2): p. 238-258.

295. Orsolini, L., et al., *Technology-facilitated sexual violence among Italian youths: validation of the technology-facilitated sexual violence victimization scale.* Frontiers in psychiatry, 2024. **15**: p. 1449183.

296. Pak, S.H.L., et al., *Measuring Technology-Facilitated Sexual Violence and Abuse in the Chinese Context: Development Study and Content Validity Analysis.* JMIR formative research, 2024. **8**: p. e65199.

297. Antoniadou, N. and C.M. Kokkinos, *Breaking the Link: Parental Attachment as a Moderator in the Relationship Between Callous-Unemotional Traits and Cyber-Bullying.* Child & Youth Care Forum, 2025. **54**(1): p. 207-225.

298. Colburn, D., K.J. Mitchell, and A. Gewitz-Meydan, *Cumulative determinants of adolescent health indicators: the effects of social and structural determinants of health and child sexual abuse on overdose and suicide attempt.* Frontiers in public health, 2025. **13**: p. 1595115.

299. Dardis, C.M., R. Prasai, and D.I. Ross, *Who Intervenes and When? Individual and Situational Correlates of Bystander Behavior When Sexual Images Are Shared Nonconsensually.* Violence against women, 2025: p. 10778012251397973.

300. Finkelhor, D., et al., *Persisting concerns about image exposure among survivors of image-based sexual exploitation and abuse in childhood.* Psychological trauma : theory, research, practice and policy, 2025. **17**(Supplement 1): p. S88-S93.

301. Gewirtz-Meydan, A., et al., *Measuring Image-Based Sexual Abuse (IBSA): Psychometric Validation and Analysis of the IBSA Scale.* Child maltreatment, 2025: p. 10775595251338188.

302. Kokkinos, C.M., T.A. Papioti, and I. Voulgaridou, *Predictors of Proclivity, Enjoyment, and Acceptance of Non-Consensual Intimate-Image Distribution Among Greek University Students.* European Journal of Investigation in Health Psychology and Education, 2025. **15**(8): p. 17.

303. Kokkinos, C.M., T. Papioti, and I. Voulgaridou, *Profiling proclivity for technology-facilitated sexual violence through Dark Tetrad traits.* Computers in Human Behavior, 2025. **165**: p. 8.

304. Pijlman, V., et al., *“I Did Not Want to Make a Bigger Deal Out of It than It Was”: A Mixed-Method Study on the Help-Seeking Behavior of Victims of Image-Based Sexual Harassment and Abuse.* Journal of Interpersonal Violence, 2025. **40**(5-6): p. 1325-1359.

305. Turner, B., *Balancing Openness and Integrity: Parliament's Role in the Age of AI and Deepfakes.* Australasian Parliamentary Review, 2025. **40**(2): p. 160-170.

306. Umbach, R. and N. Henry, *Perpetration of image-based sexual abuse in the digital age: prevalence, motivations, and community attitudes in 10 countries.* Journal of Cybersecurity, 2025. **11**(1): p. 22.

307. Maher, C.A., B.E. Hayes, and H. Liebreich, *Collegiate Lifestyle-Routine Activities and Cyberharassment and Image-Based Sexual Abuse Victimization.* Journal of School Violence, 2026. **25**(1): p. 119-132.

308. Wongsomboon, V., et al., *Experiences and correlates of cyber dating abuse in sexual and gender minority adolescent girls and feminine teens: implications for psychological wellbeing.* Psychology & Sexuality, 2025. **16**(1): p. 297-313.

309. Mathews, B., et al., *Disclosure of online child sexual victimisation: Findings from the Australian Child Maltreatment Study.* Child Abuse & Neglect, 2025. **165**: p. 13.

310. Walsh, W., et al., *Online Commercial Sexual Exploitation of Children in a National Victim Survey.* Psychological Trauma: Theory, Research, Practice, and Policy, 2025. **17**: p. 79-87.

311. Walsh, K., *A mixed-methods study of campus supports and institutional betrayal after sexual violence: Findings from a population-representative sample of U.S. college students.* Psychological trauma : theory, research, practice and policy, 2025.

312. Mathews, B., et al., *Online child sexual victimization and associated health risk behaviours and mental disorders: Findings from a national survey in Australia.* SSM - Mental Health, 2026. **9**.

313. Bhuptani, P.H., et al., *Validation of the Social Reactions-Online Questionnaire Among Adults Who Disclosed Online Victimization Via #MeToo.* J Interpers Violence, 2025. **40**(13-14): p. 3359-3377.

314. Jannite, U.K., S. Abedin, and M.M. Rahman, *Technology-facilitated sexual harassment and mental health symptoms among young-adult female student sample in Bangladesh.* Archives of Women's Mental Health, 2025. **28**(2): p. 309-319.

315. Ahn, C.K., et al., *Associations between sexting behaviours and muscle dysmorphia symptomatology among a Canadian sample of young adults.* Body Image, 2025. **55**: p. 101976.

316. Palomino-Ccasa, J., et al., *Sexting Motivation Scale (EMS) in Peruvian Youth.* Sexes, 2025. **6**(2): p. 13.

317. Ozdag, V., S. Yuce, and N. Soylu, *Online sexual abuse, sexting, and bullying among adolescents with and without ADHD: A cross-sectional study in a Turkish sample.* Acta Psychologica, 2025. **260**: p. 11.

318. Ragona, A., et al., *FoMO as a Predictor of Cyber Dating Violence Among Young Adults: Understanding Digital Risk Factors in Romantic Relationships.* Societies, 2025. **15**(9): p. 14.

319. Resett, S., P.G. Caino, and B. Mesurado, *Prediction of online grooming in adolescents based on emotional problems, sexting, cybervictimization and sex and age differences / Predicción del online grooming en adolescentes argentinos a partir de problemas emocionales, sexting, cibervictimización y diferencias de sexo y edad.* Journal for the Study of Education and Development, 2025. **48**(3): p. 721-750.
