# Supplementary material for: Measuring Technology-Facilitated Sexual Violence and Abuse: Scoping Review of Existing Measures
Source: J Med Internet Res. 2026 Apr 14;28:e90068. doi: 10.2196/90068 (PMC13125977; doi:10.2196/90068)
Supplement: Multimedia Appendix 5 [file jmir_v28i1e90068_app5.docx]

**Multimedia Appendix 5**

**Summary of validated measures (n = 55)**

| **Country** | **First author, year** | **Types of TFSVA** | **Name of measurement** | **Outcome measures** | **Scoring method** | **Timeframe** | **Targeted population** | **Validity and reliability** |
| --- | --- | --- | --- | --- | --- | --- | --- | --- |
| Spain | Borrajo, 2015 | Cyber sexual dating abuse | Cyber dating abuse questionnaire | Behavior | Likert-scale | In the past year | Adults | - EFA and CFA - Cronbach’s alpha between 0.73 – 0.87 |
| Rome | Bianchi, 2016 | Non-consensual sexting | Sexting Motivation Questionnaire | Motivation | Likert-scale | In the past year | Adolescents and young adults | - PAF and criterion validity - Cronbach’s alpha > 0.8 |
| USA | Branch, 2017 | IBSA | Perception scale on revenge porn | Attitude | Likert-scale | Not mentioned | Young adults | - Cronbach’s alpha 0.67- 0.80 |
| Sweden | Buren, 2018 | Non-consensual sexting | Sexting behavior and experiences subscale measures | Behavior | Likert-scale | Not mentioned | Adolescents | - Cronbach’s alpha 0.78- 0.85 |
| Croatia | Buric, 2021 | Non-consensual sexting | Sexting bebahvior subscale measures | Behavior | Likert-scale | In the past 6 months | Female adolescents | - Principal component analysis with parallel analysis - Cronbach’s alpha ranged form 0.65- 0.79 |
| Australia | Brown & Hegarty, 2021 | Cyber sexual dating abuse | Technology-facilitated abuse in relationships (TAR) | Behavior | Likert-scale | In the past year | Adolescents | - Construct and content validity - Cronbach’s alpha 0.91 |
| UK | Buchanan & Mahoney, 2022 | Online sexual harassment | Online Sexual Harassment Scale (OSHS) | Behavior | Likert-scale | In the past year | High school students | - CFA - McDonald’s omega 0.95 |
| USA | Bhuptani et al., 2025 | Online sexual harassment | Online Social Reactions Questionnaire (OSRQ) | Attitude | Likert-scale | Not mentioned | Adults | - Cronbach’s α = .93 and McDonald’s ω = .93 |
| USA | Choi et al., 2019 | Non-consensual sexting | Sexting involvement subscale measures | Behavior | Yes/no question | In the past year | High school students | - CFA - Cronbach’s alpha 0.74 – 0.85 |
| USA | Cornelis et al., 2020 | Non-consensual sexting | Measures related to consequences of consensual sexting | Behavbior | Likert-scale | In the past year | Undergraduate students | - Cronbach’s alpha 0.83 – 0.94 |
| USA | Cary et al., 2024 | Online sexual harassment | Online Sexual Objectification Experiences Scale | Behavior | Likert-scale | Not mentioned | Emerging adults college women | - EFA and CFA - McDonald’s omega 0.95 |
| USA | Dir et al., 2013 | Non-consensual sexting | Sexting Behavior Scale (SBS) | Behavior | Likert-scale | Not mentioned | Undergraduate students | - Convergent and discriminant validity - Cronbach’s alpha 0.81 |
| USA | Dir & Cyders, 2015 | Non-consensual sexting | Sexting Behavior Scale & Sextpectancies measures | Behavior | Likert-scale | Not mentioned | College students | - Factor analysis - Cronbach’s alpha 0.90 |
| Spain | del Rey et al., 2021 | Non-consensual sexting | Sexting Behavior and Motives Questionnaire (SBM-Q) | Behavior | Likert-scale | In the past year | Adolescent students | - EFA - Cronbach’s alpha 0.82 |
| USA | Fissel et al., 2022 | Cyber sexual dating abuse | Intimate Partner Cyber Abuse Questionnaire (IPCA-Q) | Behavior | Indicate number of times that behavior happened | In the past 6 months | Adults | - Predictive validity, convergent validity & discriminant validity - Cronbach’s alpha 0.866 |
| Spain | Gámez-Guadix et al., 2015 | Non-consensual sexting | Sexting questionnaire | Behavior | Likert-scale | Not mentioned | 18- 60 years old adults | - CFA - Cronbach’s alpha 0.78 |
| Spain | Gámez-Guadix et al., 2015 | Online sexual harassment | 10-item online victimization scale | Behavior | Likert-scale | Not mentioned | 18- 60 years old adults | - CFA - Cronbach’s alpha 0.81 |
| Spain | Gámez-Guadix et al., 2018 | Online grooming | Questionnaire for Online Sexual Solicitation and Interactions with Adults (QOSSIA) | Behavior | Likert-scale | In the past year | Adolescents between 12 and 15 years old | - Factorial analysis and concurrent validity - Cronbach’s alpha 0.69-0.87 |
| Spain | Gámez-Guadix et al., 2021 | Online grooming | Multidimensional online grooming questionnaire (MOGQ) | Behavior | Likert-scale | In the past year | Adolescents between 12 and 15 years old | - Factorial analysis and concurrent validity - Cronbach’s alpha 0.72- 0.90 |
| Spain | Gámez-Guadix et al., 2021 | Online sexual harassment | Online sexual victimization scale | Behavior | Likert-scale | In the past year | Adolescents aged 12-19 | - EFA - Cronbach’s alpha 0.82 |
| Spain | Gámez-Guadix et al., 2022 | IBSA | Image-based sexual abuse scales (IBSAS) | Behavior | Likert-scale | In the past year | Adolescents | - CFA - Cronbach’s alpha 0.65-0.93 |
| USA | Gewietz-Meydan et al., 2025 | IBSA | Image-based sexual abuse scale | Behavior | Likert-scale | Before age 18 | Young adults aged 18-28 | - EGA and CFA |
| UK | Harper et al., 2023 | IBSA | Belief and Revenge Pornography Questionnaire (BRPQ) | Attitude | Likert-scale | Not mentioned | International community sample | - EFA - Cronbach’s alpha 0.74-0.94 |
| USA | Hudson et al., 2014 | Non-consensual sexting | Sex and Tech survey instrument | Attitude and behavior | Likert-scale | In the past 30 days | Undergraduate students | - Content validity - Cronbach’s alpha 0.813 |
| USA | Hudson & Marshall, 2017 | Non-consensual sexting | Sexting measures related to behavior, negative consequences, and positive consequences | Behavior and motivation | Yes/no questions | In the past 30 days | Undergraduate students aged 18-26 | - Cronbach’s alpha 0.759-0.862 |
| USA | Hinduja & Patchin, 2021 | Cyber sexual dating abuse | Digital dating abuse victimization measures | Behavior | Yes/no questions | In the past year | American middle and high school students (12-17 years old) who have been in a romantic relationship | - EFA - Cronbach’s alpha 0.854 |
| Pakistan | Hussain et al., 2023 | Non-consensual sexting | Sexting motivation measures | Motivation | Likert-scale | Not mentioned | Women adults | - CFA - Cronbach’s alpha 0.81- 0.94 |
| USA | Klein & Cooper, 2019 | Online sexual harassment | Measure engagement in deviant cyber-sexual activities | Behvavior | Indicate number of times that behavior happened | Not mentioned | Undergraduate students | - CFA - Cronbach’s alpha 0.82 |
| Croatia | Lucić et al., 2020 | IBSA and non-consensual sexting | Sexting measures | Behavior | Likert-scale | In the past 6 months | Adolescents | - Cronbach’s alpha 0.72 |
| USA | Lopex-Cepero et al., 2021 | Cyber sexual dating abuse | Digital Intimate Partner Violence Questionnaire (DIPVQ) | Behavior | Likert-scale | Not mentioned | Adolescent students | - EFA - McDonald’s Omega > 0.70 |
| Spain | Molla-Esparza et al., 2020 | Non-consensual sexting | Adolescent Sexting Scale (A-SextS) | Behavior | Likert-scale | In the past 30 days | Secondary school pupils between ages of 11-18 | - Concurrent validity - McDonald’s omega>0.81 |
| Spain | Martínez-Bacaicoa et al., 2024 | TFSVA | Technology-Facilitated Sexual Violence Scales | Behavior | Likert-scale | In the past year | Adults | - Network analysis and solution-based exploratory factor analyses - Cronbach’s alpha > 0.708 |
| Spain | Martínez Soto et al., 2024 | Cyber sexual dating abuse | Cyber dating abuse measures (CDA victimization & CDA perpetration) | Behavior | Indicate number of incidents | In the past year | Adolescents between 14 and 18 years old | - Principal axis factoring method - Cronbach’s alpha 0.83 |
| Spain | Martínez Soto et al., 2024 | Non-consensual sexting | Sexting engagement measures | Behavior | Yes/no question | In the past year | Adolescents between 14 and 18 years old | - The principal components extraction method - Cronbach’s alpha 0.57 |
| Spain | Muñoz-Fernández et al., 2024 | Online sexual harassment | Sexual Diversity Cyberbullying  Questionnaire (SDCBQ) | Behavior | Likert-scale | Not mentioned | Adolescents | - CFA - Cronbach’s alpha 0.75-0.88 |
| Canada | Oswald et al., 2020 | IBSA | Motivations behind sharing of genital images | Motivation | Likert-scale | Not mentioned | Men adults | - Cronbach’s alpha 0.79 |
| Turkey | Ozdag et al., 2025 | TFSVA | Online sexual abuse and risky behaviors scale (JOV-Q) | Behavior | Dichotomous items (yes/no) | Not mentioned | Adolescents | - Cronbach's α =0.79 |
| Spain | Penado et al., 2019 | IBSA | Intimate Images Diffusion Scale | Behavior | Indicate number of incidents | Not mentioned | Adolescents | - CFA and concurrent validity - Cronbach’s alpha 0.976 |
| Australia | Powell & Henry, 2019 | TFSVA | Technology-facilitated Sexual Violence Victimization (TSFV-V) Scale | Behavior | Dichotomous scale | In the past year | Adults | - Cronbach’s alpha 0.93 |
| Australia | Powell et al., 2019 | IBSA | The sexual image-based abuse myth acceptance (SIAMA) scale | Attitude | Likert-scale | Not mentioned | Adults | - Cronbach’s alpha 0.86-0.94 |
| Canada | Paquette & Cortoni, 2020 | Online sexual harassment | Cognitions of Internet Sexual Offending (C-ISO) Scale | Behavior | Likert-scale | Not mentioned | Men with online and contact sexual as well as with nonsexual offences | - Convergent validity & Discriminant validity - Cronbach’s alpha 0.90 |
| Italy | Pasca et al., 2022 | Online grooming | Online Grooming Risk Scale | Behavior | Likert-scale | In the past 6 months | High school students between 13 and 18 years old | - Factorial analysis - Cronbach’s alpha 0.70-0.90 |
| Spain | Pineda-Marín et al., 2026 | Online sexual harassment | Cyber Sexual Harassment Scale-Professor Student (CSHS-PS) | Behavior | Frequency scale | Not mentioned | Adults | - Cronbach’s alpha 0.9-0.98 |
| Peru | Palomino-Ccasa et al., 2025 | Non-consensual sexting | Sexting Motivation Scale (EMS) | Motivation | Likert-scale | Not mentioned | Adults | - Aiken’s V, the minimum score was 0.8, and the maximum was 1, which is considered acceptable - EFA |
| America and China | Qu et al., 2022 | Non-consensual sexting | Sexting victimization measures | Behavior | Likert-scale | Not mentioned | College students | - Cronbach’s alpha 0.88 - Separate samples: China (0.84) and USA (0.88) |
| USA | Ritter, 2014 | Online sexual harassment | Cybersexual Harassment (CSH) Scale | Behavior | Likert-scale | Not mentioned | Graduate and undergraduate students | - EFA - Cronbach’s alpha 0.65-0.85 |
| Canada | Samimi & Alderson, 2014 | Non-consensual sexting | Sexting outcomes or behavior questions | Behavior | Likert-scale | Not mentioned | Undergraduate students aged 18 or above | - Principal component analysis - Cronbach’s alpha 0.62-0.87 |
| Spain | Sánchez et al., 2017 | Online sexual harassment | AAUW Sexual Harassment Survey for SCV & emotional distress in SCV | Behavior | Likert-scale | In the past 6 months | Secondary-level students | - EFA and CFA - Satisfactory internal consistency |
| USA | Trub & Starks, 2017 | Non-consensual sexting | Consensual sexting behavior measures | Behavior | Likert-scale | In the past 30 days | Young adults aged 18-19 | - Principle components analysis - Cronbach’s alpha 0.78 |
| Netherlands | Van Oosten & Vandencosch, 2016 | Non-consensual sexting | Willingness to engage in sexting measures | Behavior | Likert-scale | Not mentioned | Dutch adolescents (13-17 years old) and young adults (18-25 years old) | - Validity analysis - Cronbach’s alpha 0.84 |
| Belgium | Van Ouytsel et al., 2017 | Non-consensual sexting | 9-item scales that measure adolescents’ favorable attitudes towards sexting behavior | Attitude | Likert-scale | Not mentioned | Adolescents | - Principal factor analysis - Cronbach’s alpha 0.82-0.93 |
| USA | Weisskirch & Delevi, 2011 | Non-consensual sexting | Sexting Sexting Attitudes Scale | Behavior | Likert-scale | Not mentioned | Adults aged 18-30 | - EFA - Cronbach’s alpha 0.78-0.89 |
| USA | Watkins et al., 2018 | Cyber sexual dating abuse | Cyber Aggression in Relationships Scale (CARS) | Behavior | Likert-scale | In the past 6 months | Adults | - Confirmatory item factor analysis (IFA) - >80% reliability |
| USA | Yoder et al., 2018 | Non-consensual sexting | 1) Sexting behaviors  2) Sexting motivations | Behavior and motivation | Likert-scale | Not mentioned | Adolescents | - Cronbach’s alpha 0.898 |
| Egypt | Zagloul et al., 2022 | TFSVA | TFSVA measures | Behavior | Multiple choice questions | Particularly asking about the situation before and during the COVID-19 | Female adults | - Cronbach’s alpha of 80% |

**Uses of validated measures**

| **Types of TFSVA** | **Validated measurements** | **Uses of measures** |
| --- | --- | --- |
| **Non-consensual sexting** | Sexting Motivations Questionnaire (SMQ) (Bianchi et al., 2016) | 16 |
|  | Perception scale (Branch et al., 2017) | 0 |
|  | 12-items that measured sexting behavior and experiences (Buren & Lunde, 2018) | 0 |
|  | 3-item asking about sexting behaviors in the past 6 months (Buric et al., 2021) | 0 |
|  | Sexting involvement items (Choi et al., 2019) | 0 |
|  | Sexting Behavior Scale (SBS) (Dir et al., 2013) | 30 |
|  | Sexpectancies Questionnaire (Dir et al., 2013) | 3 |
|  | Expectations of sex with online-only chat partners (Drouin & Miller, 2016) | 0 |
|  | Sexting Behavior and Motives Questionnaire (SBM-Q) (del Rey et al., 2021) | 3 |
|  | Adolescent Sexting Scale (A-SextS) (Molla-Esparza et al., 2020) | 2 |
|  | Sexting questionnaire (Gámez-Guadix et al., 2015) | 15 |
|  | Sex and Tech survey instrument (Hudson et al., 2014) | 2 |
|  | Sexting questionnaire measuring attitudes towards sexting, subjective norms towards sexting, sexting behavioral intentions, and sexting behaviors (Hudson & Fetro, 2015) | 1 |
|  | Negative Sexting Consequences Scale & Positive Sexting Consequences Scale (Hudson & Marshall, 2017) | 0 |
|  | Questions related to fear of social exclusion due to sexting, gymnophobic attitudes, fear of being scammed, depression, guilt, and intention to avoid sexting (Hussain et al., 2023) | 0 |
|  | Measures about sexting (Lucić et al., 2020) | 0 |
|  | Sexting engagement measures (Martínez Soto et al., 2024) | 0 |
|  | Sexting victimization addictive scale (Qu et al., 2022) | 0 |
|  | Sexting outcomes or behaviors questions (Samimi & Alderson, 2014) | 0 |
|  | Sexting behaviors measures (Trub & Starks, 2017) | 2 |
|  | Willingness to engage in sexting (Van Oosten & Vandencosch, 2016) | 2 |
|  | Scales that measure adolescents’ favourable attitudes towards sexting behaviors (Van Ouytsel et al., 2017) | 2 |
|  | Sexting Attitude Scale (Weisskirch & Delevi, 2011) | 9 |
|  | Sexting behavior and motivations (Yoder et al., 2018) | 0 |
|  | Sexting Motivation Scale (EMS) (Palomino-Ccasa et al., 2025) | 0 |
| **Cyber sexual dating abuse** | Cyber dating abuse questionnaire (Borrajo et al., 2015) | 5 |
|  | Cyber Aggression in Relationships Scale (CARS) (Watkins et al., 2018) | 1 |
|  | Intimate Partner Cyber Abuse Questionnaire (IPCA-Q) (Fissel et al., 2022) | 0 |
|  | Digital Intimate Partner Violence Questionnaire (DIPVQ) (Lopex-Cepero et al., 2021) | 0 |
|  | Cyber dating abuse measures (Martínez Soto et al., 2024) | 0 |
|  | Digital dating abuse victimization items (Hinduja & Patchin, 2021) | 0 |
|  | TAR scale (Brown & Hegarty, 2021) | 2 |
| **Online sexual harassment/ cyber abuse/ sexuality-based cyberbullying** | Online Sexual Harassment Scale (OSHS) (Buchanan & Mahoney, 2022) | 0 |
|  | Online Sexual Objectification Experiences Scale (Cary et al., 2024) | 0 |
|  | Online sexual victimization scale (Gámex-Guadix et al., 2015; Gámex-Guadix et al, 2021) | 4 |
|  | Measure engagement in deviant cyber-sexual activities (Klein & Cooper, 2019) | 0 |
|  | Cognitions of Internet Sexual Offending (C-ISO) Scale (Paquette & Cortoni, 2020) | 0 |
|  | Cybersexual Harassment Scale | 0 |
|  | AAUW Sexual Harassment Survey for SCV & emotional distress in SCV (Sánchez, 2017) | 0 |
|  | Online sexual harassment measures (Zagloul et al., 2022) | 0 |
|  | Sexual Diversity Cyberbullying Questionnaire (SDCBQ) (Muñoz-Fernández et al., 2024) | 0 |
|  | Online Social Reactions Questionnaire (OSRQ) (Bhuptani et al., 2025) | 0 |
|  | Cyber Sexual Harassment Scale-Professor Student (CSHS-PS) (Pineda-Marín et al., 2026) | 0 |
|  | Multidimensional online grooming questionnaire (MOGQ) (Gámex-Guadix et al, 2021) | 3 |
| **Online grooming** | Questionnaire for Online Sexual Solicitation and Interactions with Adults (QOSSIA) (Gámex-Guadix et al., 2018) | 9 |
|  | Online grooming Risk Scale (Pasca et al., 2022) | 0 |
|  | Image-based sexual abuse scale (Gámex-Guadix et al, 2022) | 0 |
| **Image-based sexual abuse** | Motivations behind sending genital pictures scale (Oswald et al., 2020) | 1 |
|  | Intimate Images Diffusion Scale (Penado et al., 2019) | 1 |
|  | The sexual image-based abuse myth acceptance (SIAMA) scale (Powell et al., 2019) | 3 |
|  | Belief and Revenge Pornography Questionnaire (BRPQ) (Harper et al., 2023) | 0 |
|  | Image-based sexual abuse scale (Gewirtz-Meydan et al., 2025) | 2 |
| **TFSVA** | 21-item Technology-facilitated Sexual Violence Victimization (TSFV-V) Scale (Powell & Henry, 2019) | 7 |
|  | Technology-facilitated sexual violence scales (Martínez-Bacaicoa et al., 2024) | 1 |
|  | Online sexual abuse and risky behaviors scale (JOV-Q) (Ozdag et al., 2025) | 0 |
|  |  |  |
